# Methods for Evaluating the Effects of 2D and 3D Culture Environment on Macrophage Response to Mycobacterium Infection

**DOI:** 10.3390/microorganisms13092026

**Published:** 2025-08-29

**Authors:** Danielle L. Stolley, Komal S. Rasaputra, Elebeoba E. May

**Affiliations:** 1 Biomedical Engineering Department, University of Houston, Houston, TX 77204, USA; 2 Medical Microbiology and Immunology, University of Wisconsin-Madison, Madison, WI 53706, USA; 3 Wisconsin Institute of Discovery, University of Wisconsin-Madison, Madison, WI 53715, USA

**Keywords:** 2D versus 3D infection, structural immune response, macrophage, *Mycobacterium smegmatis*, 4D confocal imaging

## Abstract

Macrophages are critical to the formation of infection- and non-infection-associated immune structures such as cancer spheroids, pathogen-, and non-pathogen-associated granulomas, contributing to the spatiotemporal and chemical immune response and eventual outcome of disease. While well established in cancer immunology, the prevalence of using three-dimensional (3D) cultures to characterize later-stage structural immune response in pathogen-associated granulomas continues to increase, generating valuable insights for empirical and computational analysis. To enable integration of data from 3D in vitro studies with the vast bibliome of standard two-dimensional (2D) tissue culture data, methods that determine concordance between 2D and 3D immune response need to be established. Focusing on macrophage migration and oxidative species production, we develop experimental and computational methods to enable concurrent spatiotemporal and biochemical characterization of 2D versus 3D macrophage–mycobacterium interaction. We integrate standard biological sampling methods, time-lapse confocal imaging, and 4D quantitative image analysis to develop a 3D ex vivo model of *Mycobacterium smegmatis* infection using bone-marrow-derived macrophages (BMDMs) embedded in reconstituted basement membrane (RBM). Comparing features of 2D to 3D macrophage response that contribute to control and resolution of bacteria infection, we determined that macrophages in 3D environments increased production of reactive species, motility, and differed in cellular volume. Results demonstrate a viable and extensible approach for comparison of 2D and 3D datasets and concurrent biochemical plus spatiotemporal characterization of initial macrophage structural response during infection.

## 1. Introduction

Studies of immune response and disease often focus on chemical mediators of immunity. However, spatiotemporal dynamics of immune cells drive the formation of key structures such as spheroids and granulomas that are central to disease pathophysiology. Changes in chemical and structural immunity contribute to and determine progression and outcome of non-infectious diseases such as cancer, sarcoidosis, and infection-associated granulomatous diseases such as tuberculosis (TB) caused by *Mycobacterium tuberculosis* (Mtb). As the use of 3D cultures and organoids increases in prevalence, well-defined methods that compare and determine concordance between cell response in 2D versus 3D environments are needed to identify spatiotemporally dependent or independent factors that influence the formation and effectiveness of immune structures including infection-associated granulomas.

Infection-associated immune structures, the focus of this study, can occur following uptake of bacteria by primary phagocytes, triggering the production of cytokines, chemokines, and effector molecules that recruit and act on various immune cells, including neutrophils, inflammatory monocytes, interstitial macrophages, and dendritic cells, that localize to the site of infection aiding in the establishment of nascent granulomas [1,2]. The effectiveness of this structural response depends on the individual cells and their ability to coordinate a balanced response to infection. Ineffective host response such as that seen in individuals with chronic granulomatous diseases associated with defective NADPH oxidase can result in an inability of immune cells to clear bacteria like *Staphylococcus aureus*, *Burkholderia* (*Pseudomonas*) *cepacia* complex, and even *Escherichia coli*, increasing the likelihood of chronic inflammation and granulomas [3].

Within the host, dissemination of bacteria can be chemically and physically constrained through a coordinated multicellular immune response that results in extracellular matrix (ECM) remodeling, biochemical regulation of cellular interactions, and cellular aggregation [4]. Immune cells such as macrophages are key in bacterium phagocytosis, elimination, and antigen presentation. They serve as cellular bridges, centrally contributing to the modulation of biochemical and structural microenvironments during innate and adaptive immunity [5]. The ECM, also critical in structural immunity, physically sequesters inflammatory signals, oxidative molecules, and regulates proteolytic migration of macrophages and diverse immune cells [4,6]. Ineffective granulomas can lead to bacterial dissemination and concomitant reactivation of infection such as in latent TB infection (LTBI). Macrophage aggregation and nascent granuloma formation can also benefit bacteria and promote dissemination [1,7]. Characterizing the contribution of macrophages and ECMs to chemical and structural immunity is pertinent to ultimately understanding host–bacterium interaction and infection outcome in granulomatous diseases. 3D cell culture methods can be leveraged to concurrently investigate chemical and structural immune response of host–pathogen systems.

### In Vitro Models for Characterizing Granuloma Formation

In vivo infection models, ranging from zebrafish, mouse, guinea pig, rabbit, to non-human primates, combined with various cell imaging modalities have been used to characterize the structure and function of mature granulomas, as well as to investigate genetic correlates of disease, co-infection (e.g., TB-HIV), and chemotherapeutic treatments [1,7,8,9,10]. A draw-back of these in vivo models is reliance on tissue extraction and fixation to characterize early cellular interactions and precursor structures that may lead to granulomas. This sampling approach limits the ability to continuously monitor dynamic cellular interactions that drive structural innate immune response. In vitro models are often used as an alternative and are capable of replicating or mimicking 3D and 4D aspects of in vivo systems.

Mimics of nascent granulomas have been particularly important for investigating molecular to structural aspects of diseases like tuberculosis [11,12,13] and have been developed using human peripheral blood mononuclear cells (PBMCs) and mycobacterial antigen coated beads [14] as well as collagen gel embedded with fibroblasts, epithelial cells, and Mtb-infected macrophages [15]. Collagen-based microsphere aggregates of Mtb combined with human PBMCs and collagen-embedded macrophages have been used to investigate antimicrobial resistance and longitudinal immune response [16], and to develop modular microscale granuloma models, respectively [17,18]. More complex tissue mimics of infection exist, including collagen-based 3D human PBMC models of Mtb dormancy, resuscitation and anti-TNFα treatment during granuloma formation [19], and 3D models of Mtb infection in human lung mucosa, the latter of which was used to examine early phases of granuloma formation [15]. These in vitro models have successfully recapitulated biochemical and physiological features of in vivo granulomas and reproduced nutrient and oxygen gradients, cellular and cell–ECM interactions, and gene expression phenotypes observed during mycobacterial infection [20]. Extending beyond the study of spheroids and granulomas, 3D cultures are being used to develop more complex organoid systems, enabling the replication of physiologically relevant ex vivo models for studying immunopathology [21]. With advancing technology, these systems are becoming easier to rapidly produce, scalable, and personalizable using host cells. However, 3D and organoid cultures still face challenges [21], particularly in comparison to traditional 2D culture systems used in exploratory research, such as standardization, variability and reproducibility, cost, reliable sampling methods, and the need for data analysis methods to merge multidimensional and temporal data sets.

The purpose of this work is to demonstrate the feasibility of developing 3D infection models that are comparable to standard 2D culture-based models with respect to ease of implementation, relative cost, and measurement of immune response and outcomes. We develop methods to address some of the challenges of 3D systems, and characterize the impact of biological variables (antibiotics, persistence and quantifiability of fluorescent signal) and engineering constraints (oxygen diffusion and optimal sampling for 3D) on construction of 3D infection models. This work also determines areas of concordance and dissonance between macrophage infection response in 2D versus 3D environments.

## 2. Materials and Methods

### 2.1. Developing and Characterizing System and Component Constraints for the Ex Vivo Infection Platform

**Figure 1:**
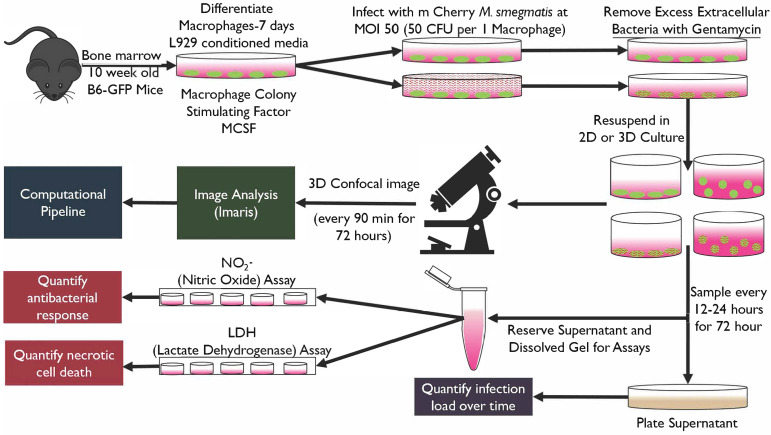
Overview of experimental methodology. To accommodate biological and engineering constraints, we developed a 3D ex vivo platform that was compatible with standard 2D sampling methods, permissive of mycobacterial infection models, and enabled imaging-based spatiotemporal monitoring. Bone-marrow-derived macrophages (BMDMs) from 10-week-old female transgenic C57BL/6-Tg (CAG-EGFP)131Osb/LeySobJ (gfpBMDM) or wild-type C57BL/6 (b6BMDM) mice (Jackson Labs) were used for all studies unless otherwise specified (isolation methods described in Supplementary Materials Section S1). Following BMDM differentiation cells were infected using mCherry expressing *M. smegmatis* strain [14] (MOI 50; 50 bacteria to 1 macrophage cell) and infected cells were resuspended in 2D or 3D culture. Infected and non-infected control cell cultures were continuously imaged (confocal) or sampled and supernatants (or dissolved gel extract) used in assays to quantify bacteria load, nitric oxide, or cell death (LDH). Experiments were conducted in replicate (*n* = 4) studies, with a minority of studies conducted in *n* = 6 replicates. A single mouse was used to conduct two technical replicates of the study.

We developed and evaluated biological and engineering constraints needed for a 3D ex vivo platform that is compatible with standard 2D sampling plus downstream assay methods, permissive of mycobacterial infection models, facilitates imaging-based spatiotemporal monitoring, and extensible for higher-throughput characterization studies (Figure 1). Bone-marrow-derived macrophages (BMDMs) from 10-week-old female transgenic C57BL/6-Tg(CAG-EGFP)131Osb/LeySobJ (gfpBMDM) or wild-type C57BL/6 (b6BMDM) mice (Jackson Labs, Bar Harbor, ME, USA) were used for all studies unless otherwise specified (isolation methods described in Supplementary Materials Section S1). We used an mCherry expressing *M. smegmatis* strain [22] to minimize spectral overlap with the gfpBMDMs during confocal imaging studies. Experiments were conducted in replicate studies (*n* = 4; two biological trials with one mouse per trial with two independent technical replicates), with a minority of studies conducted in *n* = 6 replicates. Animal studies were performed with approval of the University of Houston’s Institutional Animal Care and Use Committee (IACUC protocol no. 16-024, approved 23 September 2016) and in accordance with the Guide for the Care and Use of Laboratory Animals and the American Veterinary Medical Association (AVMA) Guidelines for the Euthanasia of Animals.

#### 2.1.1. Determining Optimal ECM Gel Height for 3D Cell Culture

To ensure balanced oxygen and nutrient diffusion, we determined ECM gel height and density constraints by taking into consideration cellular consumption rates, cell state, and duration of experiment. Using Colom et al.’s oxygen tension models (Equation (1)) for human lung epithelial cell line A549 [23] we estimated the maximum gel height based on maximal oxygen consumption rate (OCR), cellular growth rates, and assuming a heterogeneous cell population composed of non-infected, active, and infected macrophages, with active and infected cells estimated to have OCRs two and three times, respectively, the OCR of non-infected or resting cells (Table 1 and Supplementary Materials Section S1). Using the model, we approximated the maximal permissible height of reconstituted basement membrane (RBM) under non-proliferating, homogenous cell-state conditions and used the results to determine the optimal height and corresponding volume of RBM that resulted in uniformly reproducible gel layers for 3D BMDM cultures.

(1) $$h_{max}={\left(\frac{\phi_{limit}^{2}K_{m}D_{G}^{*}}{\rho_{0}V_{max}}e^{-\frac{t}{\tau }}\right)}^{1\backslash 2}$$

#### 2.1.2. mCherry *M. smegmatis* Fluorescence Homogeneity and Persistence in 2D/3D Culture

To ensure high correspondence between image-based data and standard biological assays, we isolated *M. smegmatis* strains with consistent and persistent mCherry expression via single-colony isolation and antibiotic selection methods (hygromycin 80 μg/mL, VWR, Radnor, PA, USA; Supplementary Materials Section S1, Figure S2). Infection studies were performed in the absence of hygromycin; therefore, using differential plating and microplate monitoring, we assessed plasmid persistence during extended growth in the absence of hygromycin. Briefly, mCherry *M. smegmatis* was grown in sealed (gas-permeable Breathe-Easy; Diversified Biotech, Deadham, MA, USA) black-walled 96-well plates (Eppendorf, Hamburg, Germany) in 7H9 media with and without hygromycin. Fluorescent measurements were taken every 90 min for 48 h (584 nm/612 nm, excitation/emission; FLUOstar OPTIMA BMG Labtech, Cary, NC, USA). Concurrently, static culture mCherry *M. smegmatis* was grown in filter-top glass tubes in 7H9 media with and without hygromycin for 48 h. Culture tubes were vortexed, OD600 readings taken every 24 h, and bacterial colony forming units (CFUs) enumerated by plating on 7H11 agar with and without hygromycin.

To determine the impact of the 2D and 3D environment on the bacterium, we characterized growth/death of mCherry *M. smegmatis* in 2D culture in the presence and absence of gentamicin (Sigma Aldrich, St. Louis, MO, USA) (10 μg/mL), and in 3D RBM (Sigma Aldrich), which is manufactured with a buffer solution that contains 50 μg/mL gentamicin. Bacteria in 2D culture were grown using standard 7H9 media with hygromycin (80 μg/mL) and DMEM (Dulbecco’s Modified Eagle Medium; VWR) without antibiotics to serve as a control. Samples were collected and colony forming units (CFUs) were quantified at 0, 12, 24, 48, and 72 h using 4 °C 1× PBS with 0.1% Tween 80 to disrupt the 3D extracellular matrix and remove any adhered bacteria from the tissue culture plates.

#### 2.1.3. Impact of Macrophage EGFP and Bacterial mCherry on Infection Response

Macrophage, gfpBMDM, and b6BMDM response to mCherry *M. smegmatis* infection was compared to determine potential infection response variations between wild-type and EGFP-containing cells. Using our standard 2D infection protocol, BMDMs from 8-week-old male mice were infected (MOI 50), incubated in DMEM without gentamicin, and intracellular and extracellular samples collected and replicate-plated (0, 24, and 48 h post-infection) for CFU enumeration [25]. Using the same 2D infection protocol, comparison of host response to mCherry versus wild-type *M. smegmatis* infection was conducted using b6BMDMs (MOI 50) with cells sampled in replicate (0, 24, and 48 h) for intracellular and extracellular CFU quantification.

### 2.2. Development of 2D and 3D Ex Vivo Model of Mycobacteria Infection

High-plasmid-expressing mCherry *M. smegmatis* was centrifuged at 250× *g* for 10 min and then re-suspended to desired concentration and prepared for infection in DMEM-complete (DMEM with 10% fetal bovine serum, 1% L-glutamine, and 1% non-essential amino acid). To minimize variability, a single batch of gfpBMDM cells were infected at an MOI of 1:50 host cells-to-bacteria in tissue culture plates (VWR). Supernatant was removed from culture plates and replaced with media containing bacteria. Cells were then incubated at 37 °C and 5% CO_2_ for 1 h followed by 1 h gentamicin incubation to remove extracellular bacteria [25]. Cells were washed twice with 1xPBS, detached (Cellstripper^®^, Corning, NY, USA), and placed into 2D or 3D culture.

#### 2.2.1. Infection Studies in 2D/3D Environments

The 2D ex vivo model was implemented using our standard protocol with the addition of 10 μg/mL of gentamicin in the culture media, comparable to the amount in RBM buffer. Prior studies indicated 10 μg/mL of gentamicin inhibits extracellular but not intracellular growth of *M. smegmatis* [26]. An amount of 500 μL of 2D cell suspension was added to each 24-well plate (used for sample collection) or 250 μL to non-RBM-coated wells of 8-well chamber slides (used for confocal imaging), incubated, and allowed to adhere in 2D for 2 h before sample collection.

To prevent formation of an RBM-culture plate interface void of ECM proteins we adapted Xu et al.’s protocol [27] to develop our 3D ex vivo model. We added 250 μL diluted RBM (0.18 mg/mL) coating to 48-well plates (sample collection) and 8-well (Ibidi, Fitchburg, WI, USA) chamber slides (confocal imaging), incubated (5% CO_2_ at 37 °C) for 30 min enabling matrix formation, aspirated, and removed excess RBM. Immediately following this, control (non-infected) or infected cells were resuspended in diluted 8.5mg/mL RBM (2.5 × 10^6^ cells/mL at 0–8 °C) and 100 μL of cell suspension was plated onto the center of each coated well. To minimize uneven gel dispersion and reduce meniscus formation, plates were quickly swiveled and large bubbles disrupted (20–30-gauge needle). Cell-containing matrix was incubated for 45 min, allowing gel to fully set, and 275 μL hydrating media (DMEM-complete) was added to prevent dehydration. Two independent biological experimental trials with technical replicates (total of *n* = 4 replicates) were conducted to compare 2D/3D response.

#### 2.2.2. Quantifying Biochemical Response

Supernatant samples were collected and bacterial CFUs enumerated at 0, 12, 24, 36, 48, and 72 h post-infection. For cells in 2D [25], supernatant was collected for enumeration of extracellular bacteria or downstream assays. Wells were then gently washed twice (1xPBS), 500 μL of 1% Triton-X100 added, incubated, and vigorous pipetting used to lyse cells. Lysates were vortexed, serially diluted 10-fold, and plated. After 72 h CFUs were enumerated to quantify intracellular, extracellular, or total bacterial loads.

For the 3D model, hydrating supernatant was collected and 375 μL of ice-cold PBS (0–4 °C) added and vigorously pipetted to disrupt and dissolve RBM matrix. Liquefied RBM solution was collected, centrifuged (1500 RPM, 4 °C) for 10 min, and 200 μL of solution collected for downstream assays, avoiding disruption of loose RBM/cell pellets. To disrupt cells, 200 μL of 1% Triton-X 100 was added to RBM/cell pellet, incubated (20–25 °C, 5 min), vigorously pipetted, and vortexed to lyse cells and release bacteria. Lysate was serially diluted 10-fold and plated (7H11 agar plates) for CFU enumeration.

Then, 2D/3D supernatants and 3D matrix lysates were stored at −80 °C until used to quantify nitric oxide (NO; Griess assay Promega™, G2930, Madison, WI, USA) and cell death (LDH cytotoxicity assay; Pierce™, 88954, Thermo Fisher Scientific, Fitchburg, WI, USA). Assays were performed in replicate according to the manufacturer’s protocol, with standards generated using gfpBMDMs and potential impact of RBM on the assay quantified (Supplementary Materials Section S2; Figure S3, Table S7). For 3D culture we compared sampling from the hydrating supernatant versus sampling from the intra-matrix layer, the RBM layer in which the cells are embedded. The effectiveness of 3D sampling methods was determined by comparing oxidative molecules in the intra-matrix environment (dissolved RBM) versus the supernatant (3D hydrating media; Figure S4, Tables S8 and S9). Samples from the two methods were quantified to determine significant differences between extraction methods and comparability to qualitative imaging data.

### 2.3. Image-Based Quantification of Spatiotemporal Response

Infected and non-infected cells (Ibidi 8-well) were incubated in a stage top system (TokaiHit, Fujinomiya, Shizuoka Japan) and imaged for 72 h using multi-area time-lapse confocal imaging (Olympus FV1200, UPLSAPO40X2 40×/0.95NA objective; Fluoview software v4.2b; Olympus, Center Valley, PA, USA). Two replicate wells were imaged per condition for each of the two independent trials. Images were acquired every 90 min for 100 μm Z-sections at 1 μm axial resolution (settings based on estimating Nyquist limit of macrophages and *M. smegmatis* as 10–20 μm and 2–8 μm diameter, respectively) [28,29], with dwell time set to 2 μs/pixel to reduce photobleaching and phototoxicity. The number of sample points imaged per well was determined by considering BMDMs in 2D non-infected wells (0 h) as a homogenous normally distributed population. Using the Yamane formula [30] we estimated the necessary sample size (95% CI; Equation (S1), Table S1A) and used approximations for the 2D plane to determine sample size for 3D culture. Two points per well per experimental replicate were imaged for a total of *n* = 8 imaging samples per condition, satisfying the population sampling criteria determined by the application of the Yamane formula.

#### Image Processing and Analysis

Confocal images were appended over time in Olympus Fluoview software. The resulting 4D time-appended 100 μm Z-stacks were rendered and analyzed using Imaris 8.1.2 (Bitplane, Belfast, UK) with surface creation and tracking of macrophages to enable spatiotemporal monitoring and analysis of cellular immune response. Image processing parameters (Table S1B) were condition-independent and maintained within experimental trials, with trial-specific adjustments made due to variations in background fluorescence. We analyzed 4D imaging data in time intervals comparable to biochemical sampling points (Table 2 shows total number of macrophages observed in each time interval per condition). Macrophage dynamics and features were quantified and extracted using Imaris and a Microsoft Excel-compatible feature table generated. Quantitative features include speed, acceleration, volume, displacement, and red fluorescent (RFP) values corresponding to mycobacterial load.

### 2.4. Image and Data Analysis

#### 2.4.1. Data Processing and Feature Analysis in MATLAB

We developed a MATLAB-based (MATLAB 2018b, MathWorks, Natick, MA, USA) pipeline for high-throughput analysis of the 4D structural immune response dataset. Using MATLAB GUIDE we developed an interface that facilitated data organization based on conditions (2D/3D, non-infected/infected), biological replicates, and hierarchical processing of 4D datasets. Variations in signal-to-noise ratio (SNR) were reduced post-acquisition using background normalization to bacterial RFP levels in the non-infected conditions [31]. Post-normalization, macrophage directedness (inverse of random movement) was calculated as the ratio of Euclidian distance over total distance traveled from initial to current time point, with a directedness of 1 representing straight-line movement.

#### 2.4.2. Statistical Analysis

Where noted, log_2_ fold change (Log2FC) was calculated (Equation (2)) and used for statistical comparison across experimental conditions. Rate of change values (differentials) were calculated using non-averaged data. R-Studio’s dplyr, ggplot2, ggpubr, and ggstatsplot libraries (R-Studio 1.1, 2018) were used for statistical analysis [32], with the Wilcoxon rank sum test and Pearson’s correlation used to assess statistical significance and relationships between variables.

(2) $$Log2FC=Log_{2}\left(\frac{X\left(t\right)}{X\left(0\right)}\right)$$

## 3. Results

To characterize and determine concordance of macrophage response in 3D versus 2D culture, we used a reconstituted basement membrane (RBM) to recapitulate the ECM microenvironment. We developed and used a 2D/3D ex vivo experimental and quantitative analysis platform to concurrently monitor chemical and spatial immune response during infection (Figure 1). Transgenic C57BL/6-Tg(CAG-EGFP)131Osb/LeySobJ or wild-type C57BL/6 mice (Jackson Labs) were used to generate bone-marrow-derived macrophages (BMDMs) and implement infection models consisting of green fluorescent protein expressing bone-marrow-derived (gfpBMDM) murine macrophages infected with mCherry-expressing *M. smegmatis* [22] at a multiplicity of infection (MOI; bacteria per one macrophage cell) of 50. GFP and mCherry fluorescent labels facilitated multidimensional time-lapse confocal imaging and corresponding multi-time-point sampling for downstream biochemical assays. We implemented an Imaris-MATLAB image-processing and analysis pipeline to integrate the resulting 4D spatiotemporal data with data from downstream assays measuring bacterial load and macrophage-specific biochemical response. The integrated spatiotemporal and chemical datasets were used to determine 2D/3D concordance or differences in the dynamics of structural response, production of oxidative molecules, and infection outcome.

### 3.1. Oxygen Diffusion in 3D Cell Culture Is Sufficient to Maintain Normoxia for Cell Populations

In 3D cultures sufficient oxygenation for cells in various states (infected or non-infected) is an important consideration used to determine optimal ECM gel height and density. Using Colom et al.’s oxygen tension models (Equation (1)) and based on the estimated change in maximal oxygen consumption rate (OCR) for BMDMs in RBM (5 × 10^−17^ moles/(cell (second)), we predicted maximal gel heights for maintaining normoxia and preventing cell hypoxia for non-infected, active, and infected cell populations in 3D culture (Figure S1). Non-infected macrophages maintained normoxia at a maximal gel height of 1.46 mm or approximately 140 μL of RBM per well of an eight-well chamber slide (Ibidi) or 48-well plate (VWR). Using this as an upper volumetric bound, we found 100 μL (1 mm) of RBM was sufficient to achieve a repeatable even gel layer for 3D cultures. A gel height of 1 mm enabled a homogenous population of active or non-infected BMDMs (max height permissible 1.023 mm) to maintain normoxic conditions. Although 100 μL was above our maximal estimated gel height for a homogenous culture of infected cells to maintain normoxia (maximal height of 0.822 mm), an MOI of 50 should not result in a homogenous population of infected cells throughout the duration of the study, particularly given that RBM contains an antibiotic, gentamicin. Additionally, the RBM used for calculations in the study by Colom et al. had a density of 12 mg/mL concentration, which is 50% denser than the 8.5 mg/mL concentration used in our 3D model. Therefore, we expect higher diffusivity of oxygen and nutrients as our RBM gel is less dense, allowing for greater maximal gel height. 

### 3.2. Fluorescent Strains Persist and Are Comparable to Wild Type in Growth and Infection Response

Continuous-time spatiotemporal characterization is a differentiating advantage of non-in vivo 3D and 2D infection models. However, most image-based methods require labeled cells, with some requiring antibiotics for maintenance. Potential biological variability associated with using labeled cells was assessed to ensure similar behavior of GFP/RFP and wild-type infection models and high correspondence between image-based data and standard biological assays.

#### 3.2.1. mCherry Growth and Fluorescence Maintained Absent Antibiotic Selection

**Figure 2:**
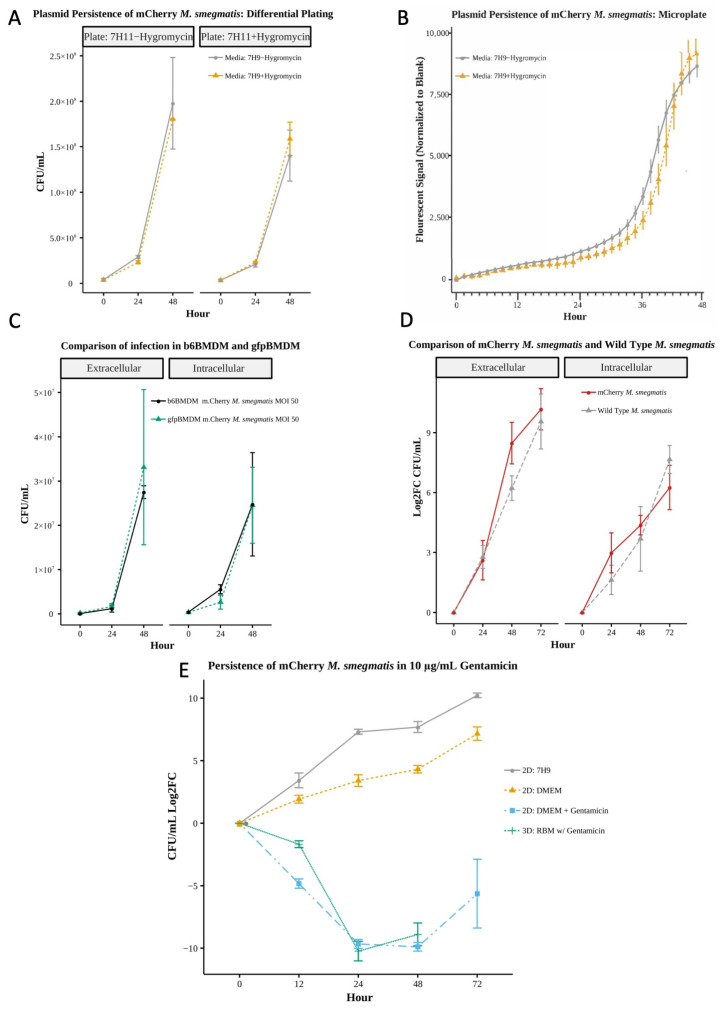
(**A**) Bacterial growth analysis represented as colony forming units at 0, 24, and 48 h of mCherry *M. smegmatis* cultured in 7H9 media in the presence or absence of selective antibiotic hygromycin subsequently plated on 7H11 agar plates with or without hygromycin. No significant difference (Wilcoxon *n* = 4). (**B**) Microplate fluorescent readings of mCherry *M. smegmatis* cultured in 7H9 media in the presence or absence of selective antibiotic hygromycin. No significant difference (Wilcoxon *n* = 6). (**C**) Comparison of gfpBMDMs and b6BMDMs under 2D infection with mCherry *M. smegmatis* at MOI 50 to determine the potential effect of GFP on macrophage–mycobacteria interaction and bacteria load. No significant difference (Wilcoxon *n* = 4). (**D**) Comparison of wild-type *M. smegmatis* and mCherry *M. smegmatis* infection in b6BMDMs. No significant difference (Wilcoxon *n* = 6, except 72 h *n* = 3). (**E**) Comparison of dynamic growth of mCherry *M. smegmatis* in 2D culture with added gentamycin at 10 μg/mL and within 3D culture of the bacterium in RBM. No significant difference (Wilcoxon testing *n* = 4). Standard media without antibiotic (DMEM) and bacterial culture media 7H9 provided for comparison. Comparison uses log_2_ (fold change (CFUs)) in order to account for variations in initial bacterial MOI.

Using differential plating (CFUs, Figure 2A, Table S2) and microplate-based fluorescent quantification (Figure 2B), we observed no statistically significant differences between mCherry *M. smegmatis* grown in media with or without hygromycin over a 48 h period in static culture. The presence or absence of hygromycin in liquid or solid culture had minimal impact on the fluorescent strains, which achieved average CFUs between 1.5–1.8 × 10^8^ with hygromycin resulting in a nominally higher fluorescent signal only after 45 h of growth. These results indicate that the purified mCherry *M. smegmatis* strain is suitable for long-term culture and the fluorescent signal is maintained and quantifiable in the absence of selective antibiotics, a necessity for imaging-based spatiotemporal monitoring of infection.

#### 3.2.2. Post-Infection Response Comparable for Fluorescent and Wild-Type Strains

Comparing macrophages from our two mice strains, we found the response of fluorescent gfpBMDMs and non-fluorescent wild-type b6BMDMs to infection with wild-type *M. smegmatis* (MOI 50) in the absence of gentamicin (Figure 2C) were comparable, exhibiting no significant differences in intracellular or extracellular bacterial load (CFUs, Table S3). Bacterial loads, with averages ranging 3.69 × 10^5^–2.48 × 10^7^ intracellular and 7.25 × 10^4^–3.31 × 10^7^, suggest that the actin-associated EGFP in gfpBMDMs did not notably interfere with macrophage–mycobacterium interaction. Similarly, there were no significant differences between mCherry and wild-type *M. smegmatis* bacterial loads post-infection of b6BMDMs (MOI 48–50 and 17–18, respectively) in the absence of gentamicin (Figure 2D, Table S4). Results indicate infection models using EGFP-tagged macrophages and mCherry bacteria are comparable to wild-type infection models, making the use of fluorescent cells viable options for implementing ex vivo infection models amenable to image-based and standard sampling-based methods for characterizing structural and chemical immune response.

#### 3.2.3. Impact of Gentamicin on 3D Bacteria Culture Reproducible in 2D Culture

To disaggregate the potential contribution of the culture environment (media or 3D matrix) on the bacteria from that of macrophage-associated immune response, we evaluated bacteria growth in culture alone without macrophage cells. The growth dynamics of mCherry *M. smegmatis* significantly differed depending on media composition and 2D versus 3D environment (Figure 2E, Tables S5 and S6). Average growth (measured as log_2_ fold change of CFUs; Log2FC) in 7H9 bacteria media was greater than in DMEM cell culture media absent gentamicin (Log2FC of 5.60 versus 3.33), with bacteria death observed in 3D RBM (Log2FC of −5.63), which contains gentamicin. However, once we add comparable gentamicin concentration (10 μg/mL) to 2D cultures to account for the presence of gentamicin in RBM, the bactericidal effect on mCherry *M. smegmatis* in 2D culture was similar to that observed in 3D RBM culture up to 48 h (average Log2FC −6.24 and −5.63, 2D versus 3D). At 72 h, the 3D culture yielded no viable CFUs, possibly due to extraction method, higher dilution factor, or low bacterial concentration. Results suggest the concentration of gentamicin used in the 2D culture is sufficiently comparable to that in 3D RBM culture. By accounting for the effect of gentamicin in 2D, we can directly investigate the structure-associated contribution of the 3D environment to infection response using a 2D/3D comparative study.

### 3.3. Extra-Matrix Sampling Sufficiently Captures Biochemical Response in 3D Infection in a Manner Comparable to Sample Collection in 2D Culture

To determine the most effective sampling method for our 3D cultures, we compared oxidative molecules sampled from dissolved RBM (intra-RBM) versus samples from the 3D supernatant or hydrating media (Figure S4, Tables S8 and S9). No significant interference from the RBM media was observed for nitric oxide (NO) standards used in the Griess reagent assay (Promega™, G2930); therefore, we determined that assays using RBM media supernatant performed comparably to standard media (Figure S3, Table S7; *n* = 5). Quantification of nitric oxide levels showed no significant difference between the collection methods, with an average supernatant NO level of 0.331 (0.412) versus intra-RBM matrix NO level 0.357 (0.377) for uninfected (infected) samples (Figure S4A, Table S8). However, the lactate dehydrogenase (LDH) cytotoxicity assay showed significant difference between samples from the intra-matrix environment versus extra-matrix supernatant for most time points (Figure S4B, Table S9), with a measured average cell death of 7.66 × 10^5^ (4.45 × 10^5^) versus 2.10 × 10^5^ (2.83 × 10^5^) quantified in uninfected (infected) supernatant versus intra-RBM matrix samples (12 to 72 h). Comparison indicated that 3D extracellular matrix supernatant from the control condition was comparable to 2D supernatant from controls for the LDH assay when comparing rate of change values (Table S18). Differences in oxidative molecules for uninfected control and infected conditions were more evident in supernatant samples. When compared to the uninfected control, infected supernatant samples on average showed a 24.4% increase in NO compared to the 5.6% increase in NO detected using intra-matrix samples. Additionally, the 3D supernatant assay results were qualitatively consistent with confocal imaging and biological observations of cellular response [25]. Results support sample collection from the extra-matrix supernatant (3D hydrating media), which is the method used in the remainder of this study to collect samples for comparing 3D and 2D biochemical response during infection. This collection method is comparable to methods used for 2D culture and amenable to high-throughput characterization studies.

### 3.4. Macrophages in 3D Culture Exhibit Similar Infection Outcomes as in 2D Culture but Biochemical Response Dynamics Differ

#### 3.4.1. Relative Increase in Oxidative Response During 3D Infection

**Figure 3:**
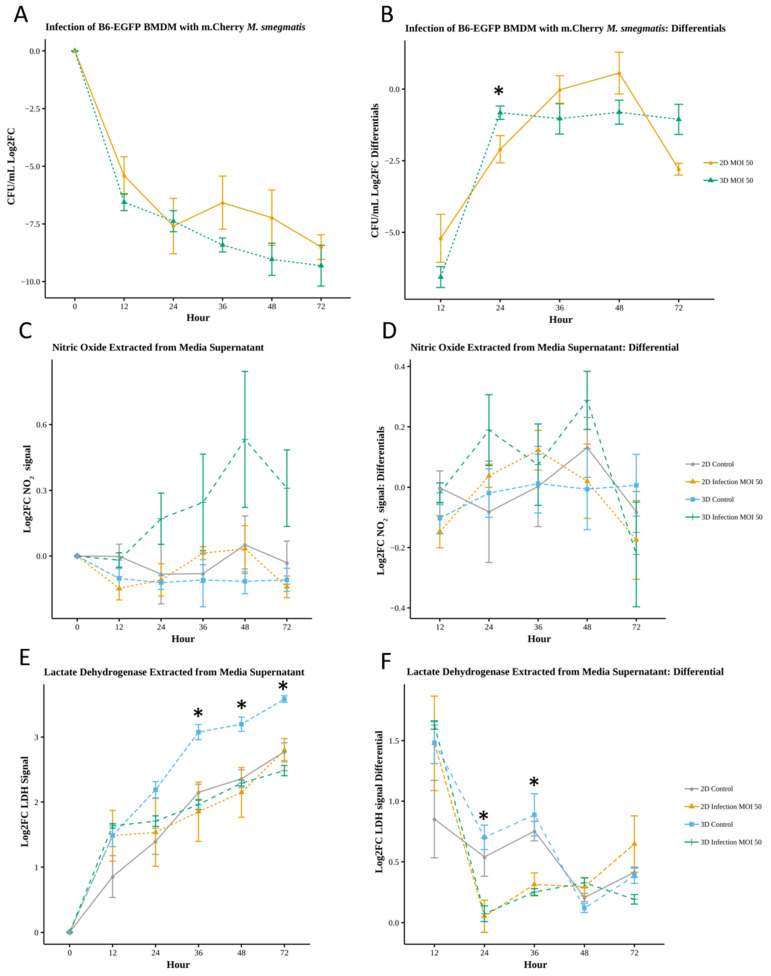
Quantification of bacterial load and effector molecules in 2D and 3D infection (* *p* < 0.05). (**A**) Change in bacterial load yields no significant difference (Wilcoxon *n* = 4). (**B**) Comparison of the rate of bacterial clearance between 2D and 3D results in significant difference (Wilcoxon *n* = 4) between 2D and 3D at 24 h only. (**C**) Nitric oxide assay yields no significant difference between any conditions. (**D**) Differential rate of change of NO signal yields no significant difference (Wilcoxon *n* = 4). (**E**) LDH assay results show significant difference between 3D control and 2D control at 36, 48, and 72 h and significant differences (*p* < 0.05) between 3D control and 3D infection at 24, 36, 48, and 72 h (Wilcoxon *n* = 4). (**F**) LDH differential shows significant difference between 2D control and 2D infection at 36 h. Significant difference (*p* < 0.05) between 3D control and 3D infection at 24, 36, and 48 h (Wilcoxon *n* = 4). Log2FC applied due to differing cell concentrations between 2D and 3D (Log2FC = log_2_ (fold change with respect to 0 h value)).

Comparison of 2D and 3D infection response showed similar bacterial clearance dynamics (Figure 3A, Table S10), with bacterial reduction in 3D averaging 15.3% higher than that in 2D (−8.14 versus −7.06 Log2FC). The average rate of change was comparable in 2D and 3D (−2.05 and −2.06 Log2FC, respectively). However, infection in 3D resulted in some qualitatively different rate of change dynamics, with 3D values significantly differing at 24 h (Log2FC of −0.82 for 3D versus −2.19 in 2D, *p* < 0.05; Figure 3B, Table S11) and positive rate of change values observed for infection in 2D (36–48 h) but not in 3D.

Comparison of NO using 2D and 3D supernatant (Log2FC) showed a general increase of nitric oxide production by macrophages in 3D infected cultures compared to 3D control, 2D control, and 2D infected cultures, with average values of 0.25, −0.11, −0.03, and −0.07, respectively (Figure 3C, Tables S12 and S13). Although not statistically significant, in comparison to their respective non-infected controls, macrophage NO production increased for 3D versus 2D cultures, with 3D infected cultures differing the most from 3D control by 0.65 Log2FC at 48 h post-infection. Similarly, the rate of change of NO was not significantly different between conditions; however, infected BMDMs in 3D had a net increased production rate of 0.06, with greatest changes in NO production at 24 and 48 h (values of 0.19 and 0.29, respectively; Figure 3D, Tables S14 and S15). We observed an increased rate of production in 2D infection conditions during the initial 36 h period, with a maximum rate of 0.12 at 36 h, followed by a decrease. Results suggest infected macrophages in 3D culture produce NO at a greater rate and differ from their 3D non-infected counterpart more than cells in 2D environments.

#### 3.4.2. Necrotic Cell Death Is Reduced for Infected Macrophages in 3D Culture

There were notable differences between the relative levels of cell cytotoxicity (LDH) for control non-infected and infected macrophages in 3D versus 2D environments. Results showed a consistent increase in macrophage death over time (Figure 3E), in concurrence with imaging observations. Differing from the NO response, control non-infected cells in 3D culture had significantly higher cell death compared to 2D control, 3D infection, and 2D infection conditions, with average Log2FC values of 2.70, 1.90, 2.01, and 1.96, respectively (*p* < 0.05, 36, 48, 72 h; Tables S16 and S17). The rate of change of LDH (Log2FC) was more comparable between control and infection conditions in 2D than 3D. In addition, 3D controls had higher relative LDH levels compared to 3D infection (*p* < 0.05, 24 to 72 h) and their rate of change differed (Figure 3F, Table S18), with infected conditions exhibiting an average 31% decrease (significant decreases between 24 to 48 h, Table S19). Infected macrophages exhibit a distinct reduction in LDH-associated cell death in 3D culture.

### 3.5. Imaging-Based Quantification Shows Differences in Macrophage 2D/3D Spatiotemporal Response

**Figure 4:**
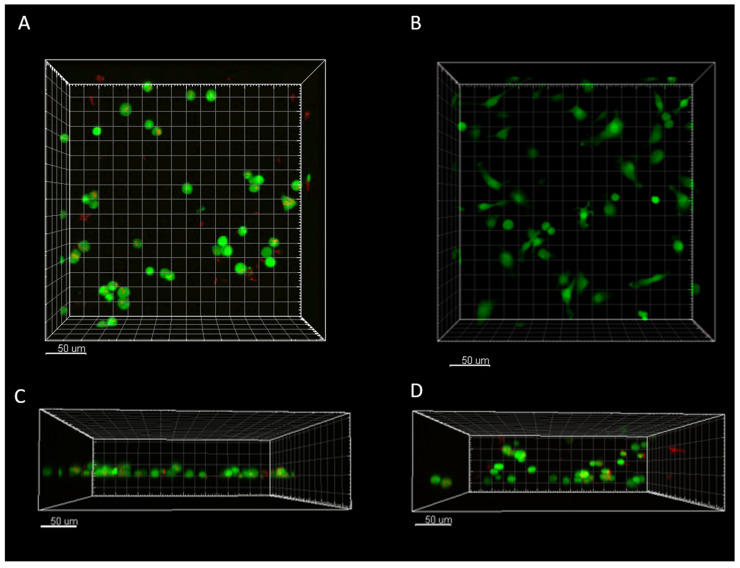
Imaris rendering of 2D and 3D infected and 2D non-infected control cells at hour 0 of imaging. EGFP expressing BMDMs are green and mCherry expressing *M. smegmatis* are red. (**A**) 2D infected macrophages demonstrating the presence of intracellular and extracellular *M. smegmatis* in infected and more rounded phenotype. (**B**) 2D control cells demonstrated more elongated and less rounded phenotype. (**C**) 2D infected macrophages side view displaying a rounded phenotype, and slight dispersion in the Z-plane. (**D**) 3D infected macrophages embedded in RBM showing dispersion through the entire imaging plane. Results and analysis of imaging data and features are presented in Figure 5, Figure 6 and Figure 7.

**Figure 5:**
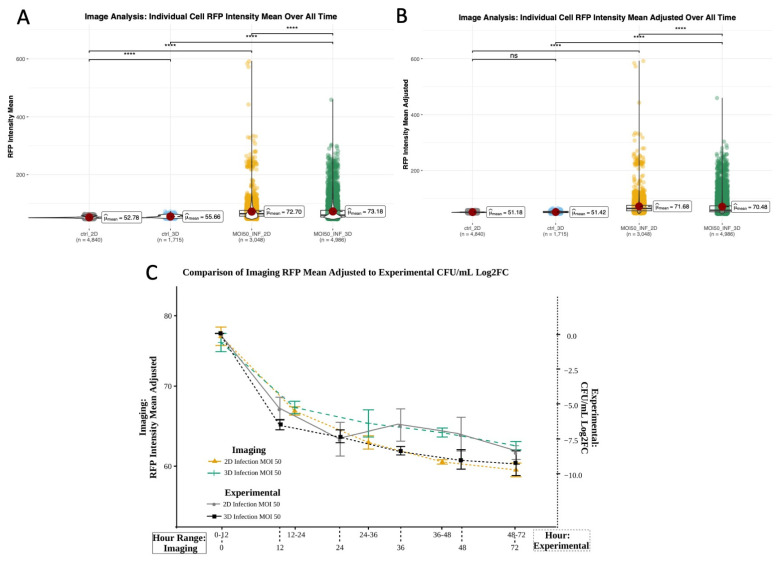
RFP mean of all cells at all timepoints (trial 1 only). (**A**) Raw RFP values. (**B**) RFP values adjusted to control RFP minimum at each timepoint (Wilcoxon ns—no significant difference, ****—*p* < 0.0001). (**C**) Overlay of adjusted RFP mean values from 4D imaging-based observations and bacterial load quantified using standard sampling and CFU plating (Log2FC CFUs/mL) shows similarity in trends over time. Imaging data were grouped into time windows that correspond to sampling time points post-infection for comparison.

**Figure 6:**
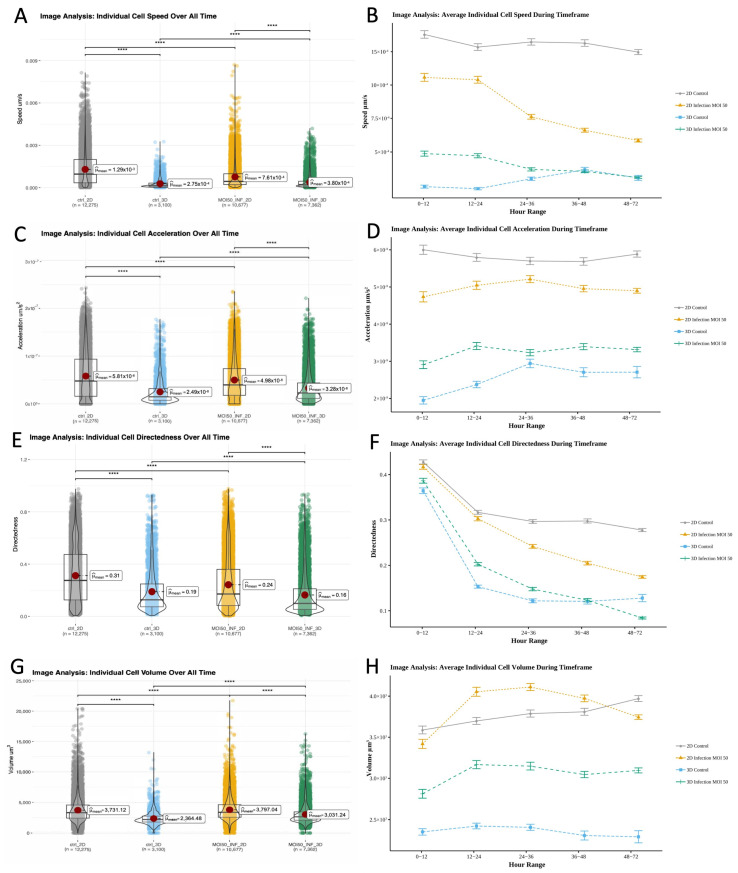
Image analysis of individual cell dynamics of gfpBMDMs infected with mCherry *M. smegmatis* in 2D and 3D culture conditions over 72 h. (**A**) Cell speed averaged over all time. (**B**) Cell speed averaged based on hour range. (**C**) Cell acceleration averaged over all time. (**D**) Cell acceleration averaged based on hour range. (**E**) Cell directedness averaged over all time. (**F**) Cell speed averaged based on hour range. (**G**) Cell volume averaged over all time. (**H**) Cell volume averaged based on hour range. (Wilcoxon, ns—no significant difference, ****—*p* < 0.0001).

**Figure 7:**
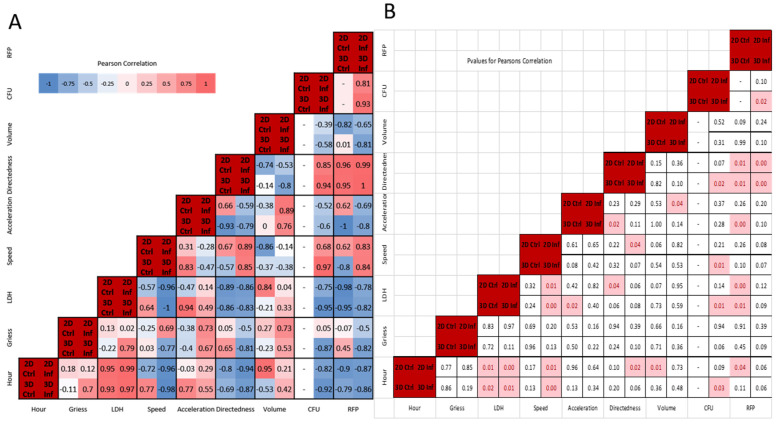
(**A**) Pearson’s correlation of all conditions. Each square includes correlation value for all conditions: upper left—2D control, upper right—2D infection, lower left—3D control, lower right—3D infection. (**B**) *p*-values for Pearson’s correlation rounded to 2 significant figures. Significant *p*-values (*p* < 0.05) are highlighted in red.

Mirroring our sample collection experiments, for each of the two biological trials (one mouse per trial), we set up two independent technical replicates of 2D and 3D culture for an imaging-based monitoring and quantification study. For each of the *n* = 4 replicates we selected two imaging regions of interest (ROIs); this yielded *n* = 8 replicate 4D image data sets per condition. Surface segmentation, quantification, and tracking analysis of the 4D data sets (Imaris) yielded a total of 33,414 unique data points (macrophage cells at all timepoints; total cells per condition shown in Table 2; images and analysis of cell features presented in Figure 4, Figure 5, Figure 6 and Figure 7), which were used to quantify 36 cell features and 20 field features representing cellular and structural characteristics of macrophage-associated immune response.

#### 3.5.1. Imaging Shows Structural and Morphological Differences Related to Infection and 3D Environment

Imaris rendering of the 4D data showed constitutive expression of actin-GFP in the BMDMs for control and infected conditions (Figure 4, see Supplementary Materials Videos S1–S4), with intracellular and extracellular mCherry *M. smegmatis* uniformly visible (Figure 4A,C,D). The 100um Z-stack sufficiently captured differences in 2D and 3D cellular distribution (Figure 4C,D), with an increase in macrophages with a rounded morphology in 2D infected versus non-infected control conditions (Figure 4A compared to 4B). Rounded morphologies have been associated with pro-inflammatory phenotypes in macrophages [33]. Cells in the 2D environment did not form perfect monolayers as macrophages can be seen in aggregates, with slight Z-plane dispersion (Figure 4C). However, Z-plane dispersion in 3D cultures was more prominent, with cells dispersed throughout the focal plane (Figure 4D) and extending well beyond the maximal imaging depth (observed under lower-resolution bright-field microscopy).

#### 3.5.2. Image-Based Characterization of 2D and 3D Cytotoxicity and Bacterial Load Shows Concordance with Standard Downstream Assays

The number of cells observed in our 4D dataset per condition concurred with LDH-based cell cytotoxicity patterns over the span of the study, with 3D control cells having the fewest number of cells. Comparison of infected to non-infected cultures reinforced results from cytotoxicity assays, with infected 2D cultures having on average fewer (87%) cells than non-infected 2D control and 3D infected cultures having markedly more (237%) macrophages than 3D control.

To minimize non-mycobacterium-associated RFP signals we compared RFPs from control (non-infected) and infected wells in 2D and 3D. RFP mean values in macrophages for the first experimental trial yielded significant differences between control and infected cells, which was expected. Evaluated over all time, raw RFP values also showed significant difference between controls (Figure 5A, Table S20), which was no longer observed post-RFP normalization to remove background RFP or bleed over from the GFP channel. As expected, post-normalization there remained significant differences in RFP values between infected and control conditions, as well as 2D and 3D infected conditions (Figure 5B, Table S21). Analysis of the adjusted RFP mean for 2D and 3D infection showed decreasing fluorescence, which qualitatively and statistically corresponded to decreased bacterial load and concurred with bacterial load measured using CFU plating over time (Figure 5C and Figure 7).

#### 3.5.3. Environment Drives Differences in Cell Motility and Migration, While Volumetric Differences Driven by Infection State

While standard and image-based quantification show that macrophages in 2D and 3D similarly clear *M. smegmatis*, to determine if there were environment-associated differences in spatiotemporal behavior or features that ultimately contribute to structure we used 4D datasets to further compare macrophage response. The fold change between overall means of individual cell speeds was greatest between 2D and 3D controls, with 2D controls having the highest overall cell speed (Figure 6A, Table 3). In 2D, the average speed of control (12.94 × 10^−4^ μm/s) was 69.5% greater (*p* < 0.0001) than infected cells (7.61 × 10^−4^ μm/s). In 3D, the inverse was observed, with infected-condition speed (3.8 × 10^−4^ μm/s) being 38.2% greater (*p* < 0.0001) than the control (2.75 × 10^−4^ μm/s) (Figure 6A, Table S22). Over time, average cell speed decreased for infected conditions (Figure 6B and Figure S5). Control 2D conditions exhibited periods of both increased and decreased speed; however, 3D control conditions mainly increased in speed. We observed significant differences (*p* < 0.0001) between all conditions for all time periods, except 3D control and 3D infected cells at 36–48 and 48–72 h (Figure 6B, Tables S23 and S24).

In 2D, the average acceleration of non-infected macrophages (5.81 × 10^−8^ μm/s^2^) was 16.7% higher (*p* < 0.0001) than 2D infected cells (4.98 × 10^−8^ μm/s^2^). Again, exhibiting the opposite behavior, the overall average acceleration of infected macrophages (3.28 × 10^−8^ μm/s^2^) in 3D culture was 31.7% higher than 3D controls (2.49 × 10^−8^ μm/s^2^, *p* < 0.0001; Table S25). These differences are clearly shown by the average and fold change acceleration values (Figure 6C, Table 4). Differences in acceleration were significant for all conditions except 2D control and 2D infected conditions during the 24–36 h period (Figure 6D and Figure S6, Tables S26 and S27).

Average cell directedness (non-random movement) decreased as macrophage cells accumulated greater total distance traveled over time. For both 2D and 3D environments, over all time points, the average directedness for non-infected macrophages (2D: 0.31165, 3D: 0.188757) was higher (*p* < 0.0001) than for infected cells (2D: 0.241949, 3D: 0.163623; Figure 6E, Table 5 and Table S28). There were significant differences between all conditions during all time frames except 2D control/infected and 3D control/infected cells for 0–12 and 36–48 h time frames, respectively (Figure 6F and Figure S7, Table S30). In 2D the directedness for non-infected conditions was greater than the 2D infected condition for every time period. However, the 3D infected condition’s average temporal directedness was 1.05% to 32.03% greater than the non-infected condition during the majority of infection when bacterial loads were higher (the 0 to 48 h window; Figure 6F, Table S29). These distinct spatiotemporal dynamics were unique to the 3D environment.

Significant volumetric differences were observed between 2D/3D and control/infected conditions throughout the study (Tables S31–S33). The average volume of macrophages in the infected condition (2D: 3797.04 μm^3^, 3D: 3031.24 μm^3^) was greater (*p* < 0.0001) than the non-infected control condition (2D: 3731.124 μm^3^, 3D: 2364.482 μm^3^; Figure 6G, Table S31), with a larger gap in volumetric fold change occurring in 3D (Table 6). During the course of the study, the volume of 2D infected macrophages initially increased to 9.37% above that of 2D non-infected cells 12–24 h post-infection, then decreased to 6% less than 2D non-infected 48–72 h post-infection. In addition, 3D conditions exhibited greater volumetric divergence (Figure 6H and Figure S8). The volume of 3D infected macrophages increased from 18.72% to 33.19% greater than that of 3D non-infected conditions, which mainly decreased in the 12–72 h post-infection window.

### 3.6. Correlative Characteristics Vary Depending on Infection and Environment

Figure 7A shows condition-specific Pearson correlations, with statistically significant correlations highlighted in Figure 7B (*p*-values < 0.05). We observed the greatest differences in temporal correlation coefficients for cell speed in 3D environments, with 0.77 versus −0.98 for 3D control versus infected. Divergent correlations in speed, volume, and marginally in NO (Greiss) also occurred for 3D conditions, with infected macrophages exhibiting positive temporal correlations for NO (0.7 versus −0.11) and volume (0.42 versus −0.53). For 2D environments most temporal correlations were in the same direction, except for acceleration, which was slightly more positive for infected conditions (0.29 versus −0.03). The greatest number of significant temporal correlations were for LDH and cellular speed (see Supplementary Materials Section S4).

#### 3.6.1. Relationship Between Cytotoxicity, Speed, and Bacteria Load Concordant in 2D and 3D Cultures, but NO Negatively Correlates with Speed and Mycobacterial Load in 3D

Consistent with previous 2D studies of *M. smegmatis*-infected macrophages (MOI 10), we noted slight positive correlations between LDH/NO and NO/CFUs in 2D [25]. The magnitude of the positive LDH/NO correlation was greater during 3D infection and, unlike the 2D environment, LDH/NO were negatively correlated in the 3D control. While the Gough et al. studies observed a positive correlation between LDH and extracellular bacteria load, we noted a significant negative correlation for both in 2D and 3D. The variation could in part be due to the MOI used in this study or the presence of gentamicin in culture media potentially reducing extracellular bacteria.

Nitric oxide was not significantly (*p* < 0.05) correlated with other measured variables. There were, however, notable environment-associated differences in correlation patterns, particularly for infected macrophages in 3D where NO and LDH were positively correlated (0.79, *p* = 0.11). Nitric oxide was also correlated with cell speed in 2D infection (0.69) but had a strong negative correlation with speed (−0.77), directedness, and CFUs in 3D infection (−0.81, −0.87; *p* ≤ 0.1). There were infection-associated differences as well, with nitric oxide positively correlated with cell volume and acceleration in both 2D/3D infected conditions (greater than 0.5), but showing minimal correlation in 2D/3D control conditions (less than +/−0.3).

LDH/cytotoxicity was negatively correlated with speed in all conditions except the 3D control (range −0.57 to −1), with significance for 2D and 3D infected conditions (−0.96, −1). LDH correlated positively with acceleration (range 0.14 to 0.94) except in the 2D control, with statistical significance in 3D controls (0.94, *p* <.05). Directedness showed strong negative correlation with LDH for all conditions (range −0.83 to −0.89), with significance in 2D controls (*p* < 0.05) and near-significance for the remaining conditions (*p* < 0.1). LDH showed strong negative correlations with adjusted RFP mean values (Figure S9) and with bacterial load/CFUs in 2D and 3D infection (−0.75, −0.95), with statistical significance in 3D infection.

#### 3.6.2. Macrophage Speed and Directedness Positively Correlate with Bacteria Load with Significance in 3D Infection Conditions

Cell speed correlated positively with directedness in 2D (0.89, *p* < 0.05) and 3D infection, and with CFUs in 3D infection (0.97, *p* < 0.05). Cellular acceleration exhibited strong negative correlation to directedness in 3D control conditions (−0.93, *p* < 0.05) and positive correlation to cell volume for both infected conditions (>0.75; *p* < 0.05 for 2D infection). CFUs and directedness had strong positive correlations under 3D infection only (0.94, *p* < 0.05).

CFUs and adjusted RFP mean were positively correlated in 2D and 3D infection (>0.8), with significance in 3D (*p* < 0.01). This correlation supports the use of RFP as representative of detectable CFU changes in 3D culture. CFUs and RFP had comparable correlation coefficients for most variables in infected 2D conditions and for all variables for infected 3D conditions, with both having negative correlations temporally, with NO (3D infected), LDH, acceleration, and volume. We observed positive correlations between CFUs/RFP and cellular speed and directedness. Temporal CFU correlations and correlations with LDH, speed, and directedness were statistically significant for 3D infection conditions only.

The positive tripartite correlation between speed, directedness, and CFUs is concordant in 2D and 3D, and indicative of directional movement driven by macrophage response to infection. It is notable that this connection is stronger in the 3D environment and only in 3D culture do the infected and non-infected states differ in the correlations between speed and directedness.

## 4. Discussion

To support movement towards ubiquitous use of in vitro and ex vivo 3D models for the study of structural immune response, we developed a platform to characterize spatiotemporal immune response and evaluated engineering constraints plus biological variables that impact model implementation and sample recovery. The platform was used to investigate concordance between 3D and 2D infection models and characterize macrophage structural immune response using GFP BMDMs infected with mCherry *M. smegmatis* to quantify oxidative response, spatiotemporal dynamics, and bacterial clearance. While overall bacterial load, nitric oxide, and LDH-based cytotoxicity were comparable in 2D and 3D infection, response relative to non-infected control conditions varied significantly for macrophages in the 3D environment.

### 4.1. Effectiveness of Biochemical Response During Infection More Pronounced in 3D Environment

Based on CFU enumeration, BMDMs in the 3D RBM environment did not differ significantly from those in 2D culture in clearance of bacteria during infection. However, the adjusted RFP mean, the imaging-based metric for bacterial load, did show environment-associated differences. Unlike 2D, infection in 3D resulted in a negative correlation between NO production and CFUs/bacteria load. This distinct correlation pattern, in addition to higher levels of NO and LDH in 3D infection and control, respectively, suggests that infection-associated production of NO is highly consequential in 3D versus 2D environments, with NO impacting macrophage cytotoxicity and bactericidal activity.

NO is also negatively correlated with LDH only in 3D control conditions. LDH has the strongest positive correlations with speed and acceleration in the 3D control, but negative correlation with directedness for all conditions. Therefore, we expect that in 3D culture, absent of infection, cell death may have a greater dependence on cell migration and proximity to other cells rather than the diffusion of oxidative molecules. The significantly higher level of LDH in 3D uninfected control further supports spatial dispersion in the 3D environment as a contributing factor to decreased cell viability.

### 4.2. 3D Spatiotemporal Response Associates with Infection and NO and Is Consistent with Prior Observations of Virulence and Proinflammatory Modulation of Cell Speed and Morphology

The overall decrease in motility in the 3D environment was anticipated due to ECM-associated impedance. However, the distinct infection-associated increase in spatiotemporal dynamics in 3D clearly indicates cellular migration and mobilization are measurably consequential in 3D versus 2D structural response to mycobacterium infection. Davis and Ramakrishnan noted increased cellular speed with increasing virulence when comparing in vivo wild-type *Mycobacterium marinum* (Mm) infection in zebrafish to infection with the less virulent ΔRD1 strain [1]. Notably, our observations of increasing macrophage speed associating with infection in 3D culture, but not 2D culture, relationally parallels the virulence-associated increase in speed observed in the wild-type versus ΔRD1 Mm zebrafish granuloma model.

The in vivo speeds observed for the Mm granuloma model (1 × 10^−2^ to 7.5 × 10^−2^ μm/s) were greater than macrophage speeds measured in our 2D and 3D models, likely due in part to the avirulent nature of *M. smegmatis.* However, our 2D control macrophage speed (1.29 × 10^−3^ μm/s) was in agreement with the cellular speed observed by Webb et al. in their 2D chemotactic studies of CSF-1-stimulated BAC1.25F murine macrophage cell line (~2.14 × 10^−3^ μm/s) [34]. Macrophage speeds for our 2D/3D infection model (7.61 × 10^−4^ μm/s, 3.8 × 10^−4^ μm/s) and 3D control (2.75 × 10^−4^ μm/s) were comparable to macrophage speeds observed for CSF-1-starved cells (~6.39 × 10^−4^ μm/s), which also exhibited a more rounded morphology similar to our 3D and infected 2D cultures.

In addition to infected versus non-infected states impacting cell motility and morphology, the macrophage population in 2D and 3D infected conditions are a heterogeneous compilation of cells [31] in various activation states ranging from pro-inflammatory (M1 type) to anti-inflammatory (M2 type) macrophages. Activation states have been shown to impact macrophage migratory patterns [35]. Cui et al. observed integrin-associated differences in 3D migration for M1 versus M2 macrophages in response to MCP-1. Similar to M1 macrophages, which Cui et al. noted had increased inducible nitric oxide synthase (iNOS) expression and more rounded morphology, relative to non-infected controls 2D and 3D infected cells in our study exhibited rounded cellular morphology, with 3D infected cells having higher NO levels than the control. Compared to the 2D control, 2D infected cells in our study had relatively lower migratory speed, which parallels the reduced migration Cui et al. observed for M1 cells with higher integrin expression. Converse to 2D infected cells, 3D infected cells initially had relatively higher speeds than their control counterpart. However, in 3D infected culture NO levels negatively correlated with speed, with NO levels increasing as cell speed decreased during the course of the infection. Our observations suggest, as observed in Cui et al., increasing NO, typically associated with M1 macrophages [35], distinctly associates with decreasing speed during *M. smegmatis* infection in 3D but not in 2D, a structure-associated discordance in macrophage immune response.

### 4.3. BMDMs in 3D Environment Exhibit Significantly Increased Volume During Infection

While direct comparison between cell volume in 2D and 3D was challenging due to differing morphologies, analysis of control versus infected 2D and 3D conditions showed a notable increase in cellular volume for infected conditions. Increased cell volume correlating to bacterial infection has been observed in granulocytes incubated with *Escherichia coli* K12, which exhibited a 52–62% increase in volume [36]. We observed a more dramatic average volume increase in 3D infected (28%) compared to 2D infected (2%) conditions. For both 2D and 3D infected conditions, volume negatively correlated with bacterial load (−0.39 and −0.58, respectively) indicative of effective phagocytosis-associated clearance of the infection particularly under 3D conditions.

### 4.4. 3D Environment Results in a Comparatively Distinct Biochemical and Structural Immune Response Signature

Macrophage response in 3D environments results in a distinct infection-associated biochemical and spatiotemporal immune signature, which is not readily observed for infected macrophages in the 2D environment. The relative concentration and impact of the measured biochemical effector was more consequential in the 3D environment. Whereas relative NO levels were minimally distinguishable in 2D infected versus non-infected cells, notably higher NO levels directly correlated to a reduction in bacteria in 3D. Correlations between NO, cytotoxicity, and bacterial load, key infection-associated phenotypes, were markedly absent in 2D but distinctly positive (NO, cytotoxicity) and negative (NO, CFUs) in 3D infection. Spatiotemporal characteristics quantified in the 3D environment exhibited a pronounced infection-associated signature, specifically for macrophage speed and volume. Compared to control non-infected conditions, correlations between speed and cytotoxicity, directedness, and time post-infection showed inverse relationships in 3D in the presence of infection. Volume-associated temporal correlations differed in 3D as did the correlations between volume, NO, and cytotoxicity.

## 5. Conclusions

We developed an integrated experimental and computational platform to compare *Mycobacterium smegmatis*-infected macrophage response in 2D versus 3D culture environments. Results show the platform’s utility, identify technical variables that influence reproducibility and integration of chemical and spatial immune response data, and show points of concordance and dissonance in macrophage infection response in 2D versus 3D culture. While macrophages in 2D and 3D cultures were comparable in mycobacteria clearance and correlations between spatiotemporal movement and bacteria load, there were environment-associated differences. We observed macrophages in 3D environments increased production of reactive species (nitric oxide), motility, and differed in cellular volume. Also, stronger correlations and distinction in non-infected/infected spatiotemporal response were more distinct in 3D versus 2D culture.

This work demonstrates the importance of the ECM and physiological components of immune response. Our results suggest some concordance in the outcome of infection for 2D and 3D environments, but notable discordance on the effects of NO and divergence between non-infected and infected cells in 3D versus 2D environments. Several differentiating features observed in 3D concurred with observations from in vitro migration studies and in vivo infection studies with mycobacteria of varying virulence. Outcomes demonstrate the feasibility of using traditional sample collection plus 4D confocal imaging methods to concurrently probe 3D biochemical and structural immune response to infection using cost-effective culture methods. Methods used are extensible to studies with pathogenic mycobacterium and inclusion of more complex combinations of innate immune cell populations, which will be required to fully characterize structural immunity, nascent granuloma formation, and function in infection clearance, containment, and dissemination.

## Figures and Tables

**Table 1:** Colom et al.’s formula for maximal gel height to prevent hypoxia in RBM culture.

Variable	Description and Values Used
$h,h_{max}$	(Maximum) gel thickness
$\phi_{limit}$	Thiele modulus for normoxic environment (19 in for half gel depth, 16.45 for full gel depth (estimate)) assuming an oxygen tension limit of 50 mmHg [23]; normoxic oxygen tension is 20 to 50 mm Hg in most tissues (excluding high oxygenated environments such as arteries and alveoli)
$K_{m}$	Oxygen concentration at which OCR is half of Vmax; $V_{max}$ (0.45 mmHg = 0.58 × 10^−3^ mol m^−3^) [23]
$D_{G}^{*}$	Oxygen diffusivity in the gel in the presence of cells (1.7 × 10^−5^ cm^2^/s, graphical estimation) [23]
$p_{0}$	Initial cell seeding density (2.5 × 10^6^ cells/mL)
$V_{max}$	Maximum OCR per cell Maximum OCR for A549 in gel is ~3/10ths of 2D OCR (graphical estimation) [23] Maximum OCR for BMDMs in 2D culture is ~1.7 × 10^−16^ moles/(cell (second)) [24], which equates to an estimate of 5 × 10^−17^ moles/(cell (second)) for BMDMs in 3D RBM culture
τ	Time constant describing cell proliferation rate (54 h for A549 cells in gel) [23]; however, primary BMDMs do not proliferate well and attempts to extract cells from gel after 24 h produce fewer cells than seeded, so the exponential $e^{-\frac{t}{\tau }}$ term will trend towards 1, changing the cell growth term from $p_{0}e^{\frac{t}{\tau }}$, an assumption of exponential growth, to $p_{0},\;$an assumption of no cell growth
*t*	Time in cell culture (hours)

**Table 2:** Number of observations (cells) during each hour-range.

Hour-Range	2D Control	3D Control	2D Infection MOI 50	3D Infection MOI 50
**0–12**	1586	740	1205	959
**12–24**	2171	855	1779	1309
**24–36**	2502	718	2044	1341
**36–48**	2337	453	2107	1452
**48–72**	3679	334	3542	2301
**Total**	12,275	3100	10,677	7362

**Table 3:** Average individual cell speed over all time: log_2_ fold change of means.

Condition 1	Condition 2	Log_2_ Fold Change
2D control	3D control	2.235
2D infection	3D infection	1.0015
2D control	2D infection	0.766
3D control	3D infection	−0.467

**Table 4:** Average individual cell acceleration over all time: log_2_ fold change of means.

Condition 1	Condition 2	Log_2_ Fold Change
2D control	3D control	1.223
2D infection	3D infection	0.601
2D control	2D infection	0.224
3D control	3D infection	−0.389

**Table 5:** Average individual cell directedness over all time: log_2_ fold change of means.

Condition 1	Condition 2	Log_2_ Fold Change
2D control	3D control	0.723
2D infection	3D infection	0.564
2D control	2D infection	0.365
3D control	3D infection	0.206

**Table 6:** Average individual cell volume over all time: log_2_ fold change of means.

Condition 1	Condition 2	Log_2_ Fold Change
2D control	3D control	0.658
2D infection	3D infection	0.325
2D control	2D infection	−0.025
3D control	3D infection	−0.357

## Data Availability

The datasets presented in this article are not readily available because dataset is part of an ongoing study. Requests to access the datasets should be directed to the corresponding author (emay5@wisc.edu).

## Supplementary Materials

The following supporting information can be downloaded at https://www.mdpi.com/article/10.3390/microorganisms13092026/s1: Figure S1: Quantification of maximal RBM height; Figure S2: Purificaiton of mCherry *M. smegmatis*; Figure S3: Comparison of Griess assay standards between standard media, and media supernatant cultured on top of 3D RBM; Figure S4: Comparison of Nitric Oxide and LDH From Media supernatant and RBM; Figure S5: Image analysis of individual cell speed of gfpBMDM infected with mCherry *M. smegmatis* in 2D and 3D culture; Figure S6: Image analysis of individual cell acceleration of gfpBMDM infected with mCherry *M. smegmatis* in 2D and 3D culture; Figure S7: Image analysis of individual cell directedness of gfpBMDM infected with mCherry *M. smegmatis* in 2D and 3D culture; Figure S8: Image analysis of individual cell volume of gfpBMDM infected with mCherry *M. smegmatis* in 2D and 3D culture; Figure S9: Image analysis of individual cell RFP Intensity mean (adjusted) of gfpBMDM infected with mCherry *M. smegmatis* in 2D and 3D culture; Table S1A: Yamane formula for determination of sample size; Table S1B: Image Processing Parameters for Imaris; Table S2: Plasmid Persistence of mCherry *M. smegmatis*; Table S3: 2D MOI 50 Comparison of b6BMDM and gfpBMDM mCherry *M. smegmatis*; Table S4: 2D MOI Comparison of mCherry *M. smegmatis*: and wild type *M. smegmatis* infection in b6BMDM; Table S5: 2D 3D persistence of m.Cherry *M. smegmatis* in 10 µg/mL Gentamycin; Table S6: 2D static growth of m.Cherry *M. smegmatis* in DMEM and 7H9 Media; Table S7: Comparison of nitric oxide standard in culture media and media cultured on top of RBM; Table S8: Griess Assay-Signal Extracted from Media Supernatant and RBM; Table S9: LDH Assay Cell Death per mL Extracted from Media Supernatant and RBM; Table S10: 2D 3D MOI 50 CFU/mL; Table S11: 2D 3D MOI 50 differentials; Table S12: Nitric Oxide Extracted from Media Supernatant 2D vs. 3D; Table S13: Nitric Oxide Extracted from Media Supernatant Control vs. Infection; Table S14: Differentials-Nitric Oxide Extracted from Media Supernatant 2D vs. 3D; Table S15: Differentials—Nitric Oxide Extracted from Media Supernatant Control vs. Infected; Table S16: LDH Extracted from Media Supernatant 2D vs. 3D; Table S17: LDH Extracted from Media Supernatant Control vs. Infected; Table S18: Differentials-LDH Extracted from Media Supernatant 2D vs. 3D; Table S19: Differentials-LDH Extracted from Media Supernatant Control vs. Infected; Table S20: RFP intensity mean Trial 1 -not adjusted; Table S21: RFP intensity mean Trial 1 adjusted; Table S22: Average Individual Cell Speed Over All Time; Table S23: Average Individual Cell Speed 2D vs. 3D Over Time (timeframes); Table S24: Average Individual Cell Speed Control vs. Infected Over Time (timeframes); Table S25: Average Individual Cell Acceleration Over All Time; Table S26: Average Individual Cell Acceleration 2D vs. 3D Over Time (timeframes); Table S27: Average Cell Acceleration Control vs. Infected Over Time (timeframes); Table S28: Average Individual Cell Directedness Over All Time; Table S29: Average Individual Cell Directedness 2D vs. 3D Over Time (timeframes); Table S30: Average Cell Directedness Control vs. Infected Over Time (timeframes); Table S31: Average Individual Cell Volume Over All Time; Table S32: Average Individual Cell Volume 2D vs. 3D Over Time (timeframes); Table S33: Average Individual Cell Volume Control vs. Infected Over Time (timeframes); Video S1: Non-infected gfpBMDMs in 2D (A_2D_ctrl_1_2); Video S2: gfpBMDMs infected with mCherry *M. smegmatis* in 2D (A_2D_msmeg_1_1_01); Video S3: Non-infected gfpBMDMs in 3D (A_3D_ctrl_1_2_01); Video S4: gfpBMDMs infected with mCherry *M. smegmatis* in 3D (A_3D_msmeg_1_1_01) [37,38,39,40].
