# Supplementary material for: Methods for Evaluating the Effects of 2D and 3D Culture Environment on Macrophage Response to Mycobacterium Infection
Source: Microorganisms. 2025 Aug 29;13(9):2026. doi: 10.3390/microorganisms13092026 (PMC12472957; doi:10.3390/microorganisms13092026)
Supplement: Supplementary file 1 [file microorganisms-13-02026-s001.zip › Supplementary Materials 1.pdf]

Methods for Evaluating the Effects of 2D and 3D Culture Environment on Macrophage Response to Mycobacterium Infection

Danielle L. Stolley[1], Komal S. Rasaputra[1], Elebeoba E. May[1,2,3,*]

[1]Biomedical Engineering Department, University of Houston, USA

[2]Medical Microbiology and Immunology, University of Wisconsin-Madison, USA

[3]Wisconsin Institute of Discovery, University of Wisconsin-Madison, USA

Supplementary Materials

1. Supplemental Methods

Isolation and storage of bone marrow derived macrophages for infection assay

Bone marrow from 10-week-old female transgenic C57BL/6-Tg(CAG-EGFP)131Osb/LeySobJ (gfpBMDM) or C57BL/6 (b6BMDM) mice (Jackson Labs) used to generate bone marrow derived macrophages for infection studies were isolated as previously described [1]. Extracted bone marrow was subsequently aliquoted into freezing medium and stored in liquid nitrogen for up to four months according to the protocol outlined by Marim et al. [2]. One week prior to experiment start, aliquots were removed from liquid nitrogen, thawed and cultured as previously reported to differentiate the isolated bone marrow into macrophages [1].

Maximal RBM height permissible to prevent hypoxia of BMDM

The introduction of bacterial infection results in a heterogeneous cell population that includes resting, active (cells producing and responding to a proinflammatory environment), and infected macrophages, with active and infected cells presumably having a higher OCR than their resting counterparts (estimated as two and three times the OCR of resting cells, respective) [4,5]. Using

these assumptions, we approximate the maximal height of RBM permissible under non-proliferating, homogenous cell-state assumptions. Cell cultures composed of active cells (max height permissible 1.023 mm) are still under normoxic conditions at 100 µL of RBM at a 1 mm gel, but a homogenous cell culture composed of infected cells (maximal height of 0.822 mm) is not (Figure S 1).

The RBM used in the study by Colom et al. was 12 mg/mL concentration, 50% denser than the 8.5 mg/mL concentration for our 3D model. Using 140 µL as an upper limit, we tested various volumes to find the minimal volume necessary to achieve a repeatable even gel layer, resulting in a 100uL RBM volume for 3D culture in 8 well chamber slides (1mm RBM height), sufficiently below the normoxic limit for resting OCR for non-infected b6BMDMs.

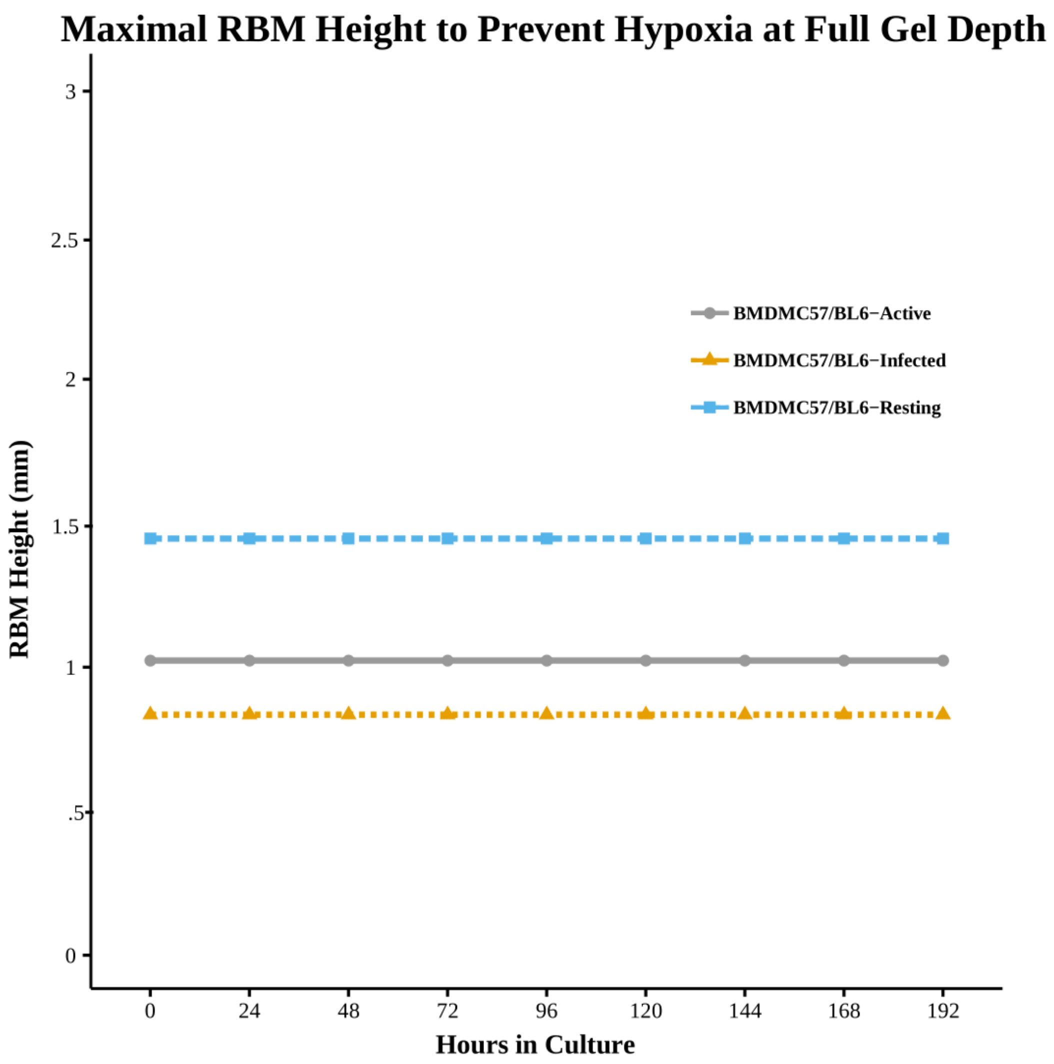

Figure S 1 Quantification of maximal RBM height to prevent hypoxia in 3D culture with assumptions of macrophage state, Active, Infected and Resting according to computational approximations of oxygen consumption rate.

m. Cherry M. smegmatis purification of expression

The accurate quantification of bacterial load via confocal imaging depends on the consistent ubiquitous expression of m. Cherry within each bacterium. To ensure even fluorescence bacterial stocks were streaked for singles on 7H11 plates with 80 µg/mL of hygromycin, the highest expressing CFU's were selected for (both colormetric and fluorescent) and subsequently streaked for singles until all colonies displayed a consistent level of m. Cherry expression. A single colony was then used to inoculate liquid culture which was then grown into the log phase (37 C at 250 RPM). The liquid stock was then plated to check for m. Cherry expression and used to make the working frozen aliquots of *m. Cherry M. smegmatis*.

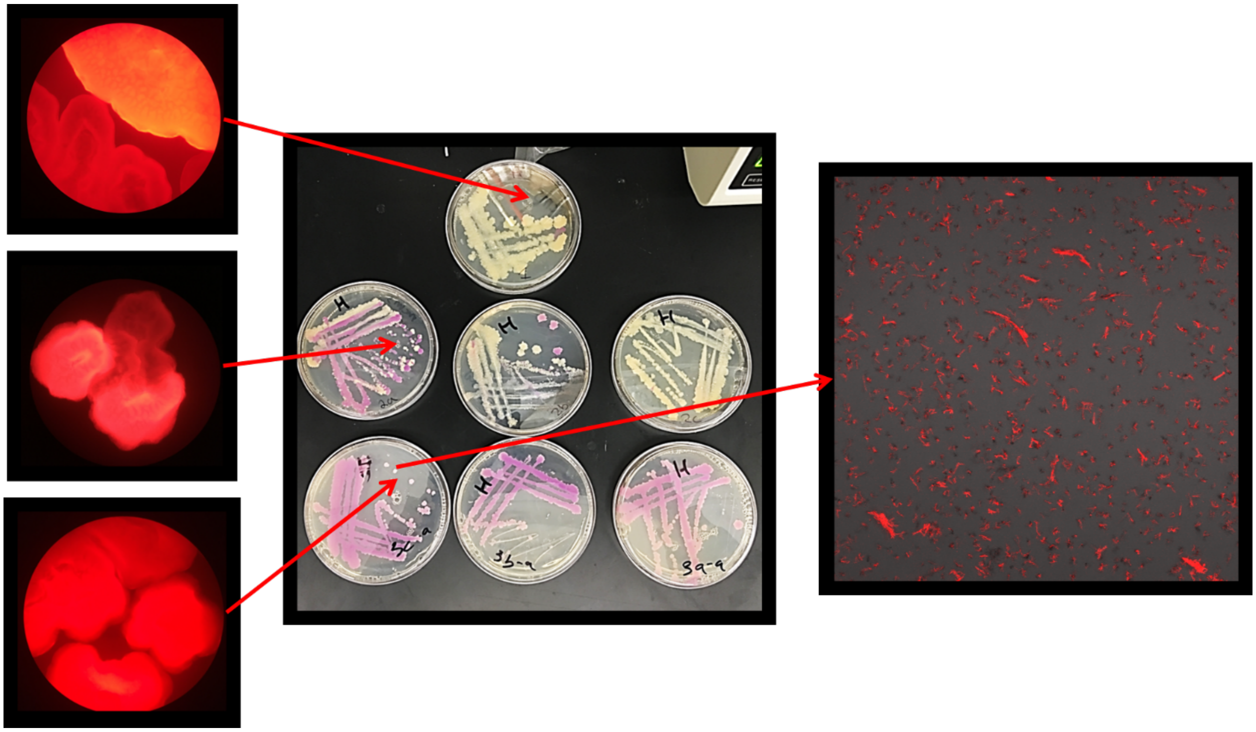

Figure S 2: Purificaiton of mCherry M. smegmatis thorugh single colony expansion and isolation of highest flourescent mCherry expressors.

Imaging Sample Size Determination and Imaris Image Processing Parameters

The Yamane formula was used to estimate the necessary sample size (95% CI; Equation S1, Table S1A) and used approximations for the 2D plane to determine sample size for 3D culture.

$$n_o = \frac{z^2 p(1-p)N}{z^2 p(1-p)N\epsilon^2} \tag{S1}$$

Table S1A: Yamane formula for determination of sample size in a homogenous normally distributed population

Variable	Description and Values Used
$\boldsymbol{n_o}$	Sample size (5E5 cells/mL * .250 uL)
$\boldsymbol{z}$	Confidence interval (1.96 for 95% confidence interval)
$\boldsymbol{p}$	Population proportion (.5)
$\boldsymbol{N}$	Population size (1.25E5 cells per 8 well chamber slide in 2D; 5E5 cells/mL * .250 mL)
$\boldsymbol{\epsilon}$	Error limit (0.03)

Table S1B: Image Processing Parameters for Imaris *(*values adjusted for each trial due to variations in fluorescence between experiments)*

	Parameter	Value
**Algorithm**	Enable Region of Interest	False
	Enable Region Growing	True
	Enable Tracking	True
**Source Channel**	Source Channel Index	1 (GFP)
	Enable Smooth	True
	Surface Grain size	1.00 μm
	Enable Eliminate Background	True
	Diameter of Largest Sphere	10.0 μm
**Threshold**	Enable Automatic Threshold	False
	Manual Threshold Value	*
	Active Threshold	True
	Enable Automatic Threshold B	True
	Manual Threshold Value B	*
	Active Threshold B	False
	Region Growing Estimated Diameter	8.25 μm
**Classify Seed Points**	Quality	Above 40.0
	Distance to Image Border XYZ	Above 3 μm
	Intensity Mean Ch=1	Above *

	Intensity StdDev Ch=1	Below *
**Classify Surfaces**	None applied	-
**Tracking**	Algorithm Name	Autoregressive Motion
	MaxDistance	20.0 μm
	MaxGapSize	0
	Fill Gap Enable	true
**Classify Tracks**	None applied	-

2. Supplemental Results –Biological Components, Environment, and Sampling

Fluorescent strains persist in culture and exhibit comparable phenotype as wild type

Table S 2: Plasmid Persistence of mCherry *M.smegmatis* Differential Plating: Wilcoxon (n=4)

Hour	*Media:+Hygro/Plate:+Hygro CFU/mL*	*Media:-Hygro/Plate:+Hygro CFU/mL*	*p value*
**0**	2.28E+07	2.43E+07	0.665006
**24**	2.28E+07	2.43E+07	1
**48**	1.50E+08	1.53E+08	0.746886
Hour	*Media:+Hygro/Plate:-Hygro CFU/mL*	*Media:-Hygro/Plate:-Hygro CFU/mL*	*p value*
**0**	2.29E+07	2.57E+07	1
**24**	2.29E+07	2.57E+07	0.060602
**48**	1.83E+08	1.80E+08	1

Table S 3: 2D MOI 50 Comparison of b6BMDM and gfpBMDM mCherry *M. smegmatis*: Wilcoxon (n=4)

Hour	*b6BMDM: Intracellular CFU/mL*	*gfpBMDM: Intracellular CFU/mL*	*p value*
**0**	4.38E+05	3.69E+05	0.465124
**24**	5.56E+06	2.66E+06	0.312321
**48**	2.48E+07	2.45E+07	0.880933
Hour	*b6BMDM: Extracellular CFU/mL*	*gfpBMDM: Extracellular CFU/mL*	*p value*
**0**	7.25E+04	2.04E+05	0.771503
**24**	1.21E+06	1.73E+06	0.665006
**48**	2.75E+07	3.31E+07	0.303525

Table S 4: 2D MOI Comparison of mCherry *M. smegmatis*:and wild type *M. smegmatis* infection in b6BMDM Log2FC Wilcoxon (n=6,*n=3). *Comparison uses Log₂(Fold Change (CFU)) in order to account for variations in initial bacterial MOI.*

Hour	*mCherry: Intracellular log2FC*	*wild Type: Intracellular log2FC*	*p value*
**0**	0	0	-
**24**	2.980128	1.626583	0.29795
**48**	4.370521	3.684769	0.81018
**72**	6.258945	7.662005	0.38273*
Hour	*mCherry: Extracellular log2FC*	*wildType: Extracellular log2FC*	*p value*

**0**	0	0	-
**24**	2.614937	2.773342	1
**48**	8.478391	6.226576	0.09270
**72**	10.17503	9.55832	0.66252*

Quantification of the impact of the environment

To determine the impact of 2D and 3D environments on the bacterium, we characterized growth/death of mCherry *M. smegmatis* in 2D culture with and without gentamicin and compared results to growth in 3D RBM.

Table S 5: 2D 3D persistence of m.Cherry *M.Smegmatis* in 10 µg/mL Gentamycin: Log2FC Wilcoxon (n=4)

Hour	*2D-Gentamycin*	*3D-RBM*	*p value*
**0**	0	0	-
**12**	-5.419	-3.410	0.245278
**24**	-9.658	-10.232	0.665006
**48**	-9.889	-8.896	0.312321
**72**	-5.633	n/a	n/a

Table S 6: 2D static growth of m.Cherry *M.Smegmatis* in DMEM and 7H9 Media: Log2FC Wilcoxon (n=4)

Hour	*2D-DMEM*	*2D-7H9*	*p value*
**0**	0	0	-
**12**	1.741	2.788	0.245278
**24**	3.409	7.316	0.030383
**48**	4.312	7.687	0.030383
**72**	7.166	10.226	0.030383

LDH standard curve

An LDH standard curve (LDH cytotoxicity assay; Pierce™, 88954) was generated through quantification of lysed concentrations of 5e6, 5e5, 5e4, 5e3, 5e2 gfpBMDMs per mL cultured in

a 24 well plate (VWR) according to manufacturer protocol. Standard curve was generated in RStudio using linear model fitting function *lm*. to generate,

$$Dead\ Cells/mL = (\mathrm{LDH_{signal}})1299870 - 191580 \quad \text{(S1)}$$

Griess Assay-Nitric Oxide standard in media and RBM media supernatant

In addition, we quantified any potential interference due to the presence of RBM in 3D culture by conducting a comparison between DMEM-complete plus 10 µg/mL of gentamicin and DMEM-complete cultured on top of RBM for a Griess reagent standard curve. The RBM Media Supernatant was compared to standard media to determine any assay interference in the formation of nitric oxide standards according to manufacture protocol Griess reagent (Promega™, G2930). No significant difference was found between the two conditions (n=5).

Table S 7: Comparison of nitric oxide standard in culture media and media cultured on top of RBM (Wilcoxon n=5)

NO Concentration	*DMEM-Signal*	*RBM-Media Supernatant-Signal*	*p value*
**100**	0.755667	0.706333	0.19043
**50**	0.537667	0.496	0.080856
**25**	0.413	0.390333	0.382733
**12.5**	0.367333	0.359	1
**6.25**	0.332667	0.329333	1
**3.13**	0.294333	0.314333	0.662521
**1.56**	0.313333	0.335	0.662521
**0**	0.302	0.322333	0.080856

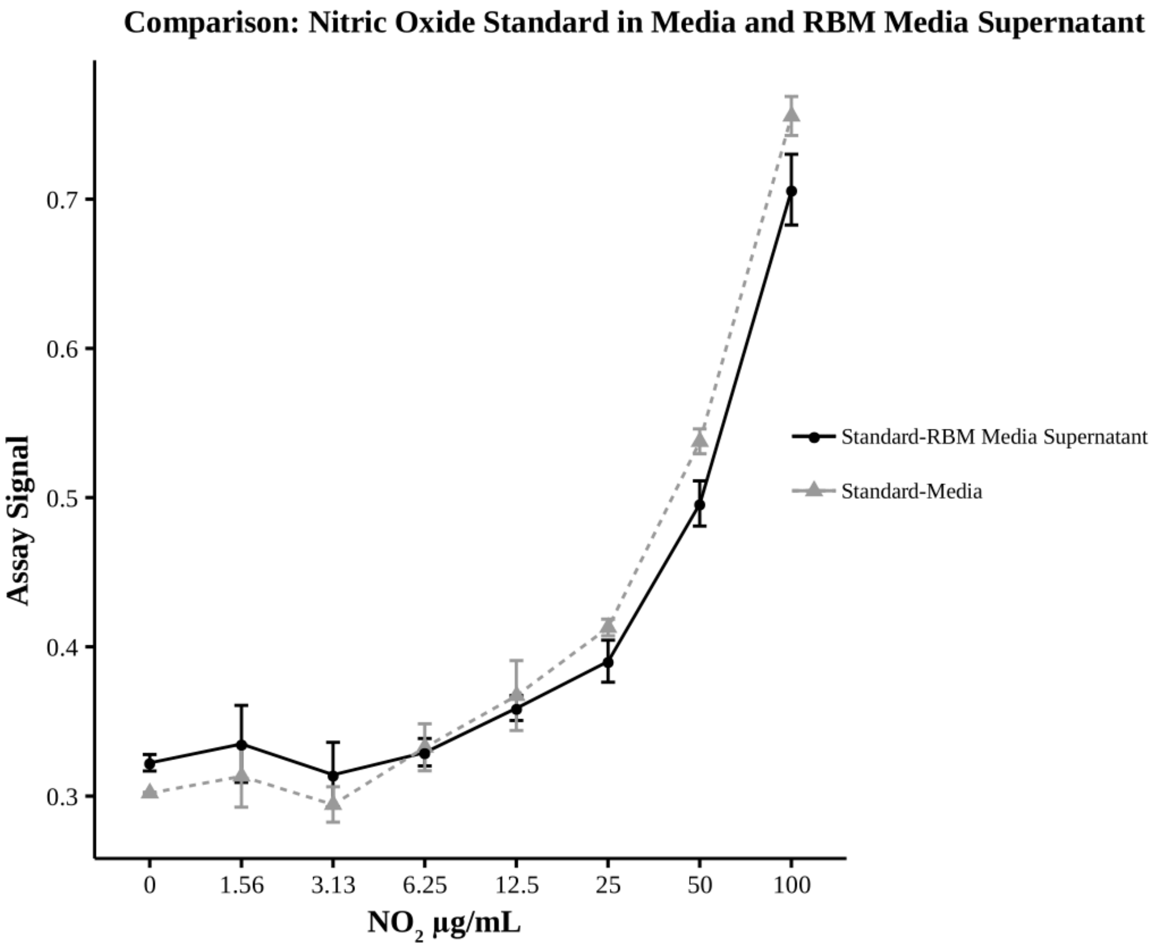

Figure S 3: Comparison of Griess assay standards between standard media, and media supernatant cultured on top of 3D RBM culture.

Determination of method for effector molecule quantification

Standard 2D cell culture and infection assays rely on the media supernatant to run immunological assays to determine effector molecule expression in response to stimulus. However, in the case of 3D studies, the hydrating supernatant is separated from intra-matrix cells by the RBM layer in which the cells are embedded. To determine the difference between the intra-matrix environment versus the supernatant and to determine a feasible method for quantifying the effector response we quantified two methods of sample extraction to determine significant differences and comparability to qualitative imaging data. We compared two methods, sampling the hydrating supernatant surrounding the RBM-cell suspension or the supernatant from disrupted RBM (disrupted using 1xPBS at 4˚C) for sample extraction. The disrupted RBM is significantly diluted during this process leading to potential loss of signal even when the dilution is accounted for. The Griess assay for quantification of nitric oxide expression showed no significant difference between the two sample collection methods for 3D. However, this is likely attributed to the already very low signal from this assay (Figure S4A, Table S8). The LDH assay showed significant difference between intra-matrix versus extra-matrix supernatant for almost all time points (Figure S4B , Table S 9). In addition, the dynamics of the media hydrating supernatant sample matched most closely to qualitative biological observations of confocal imaging and the dynamical trends for 2D, and previous studies of low level mycobacterial infection[3]. Therefore, we proceeded to compare 2D supernatant assay samples with 3D media supernatant assay samples. Comparisons of 3D extra-matrix supernatant (sample from hydrating media) versus intra-matrix (sample from dissolved RBM) supernatant indicated that 3D extra-matrix supernatant from control conditions was comparable to 2D supernatant from controls for

the LDH cytotoxicity assay. Additionally, the assay results were qualitatively consistent with confocal imaging and biological observations, therefore extra-matrix supernatant is used for comparing 3D and 2D nitric oxide and LDH assays.

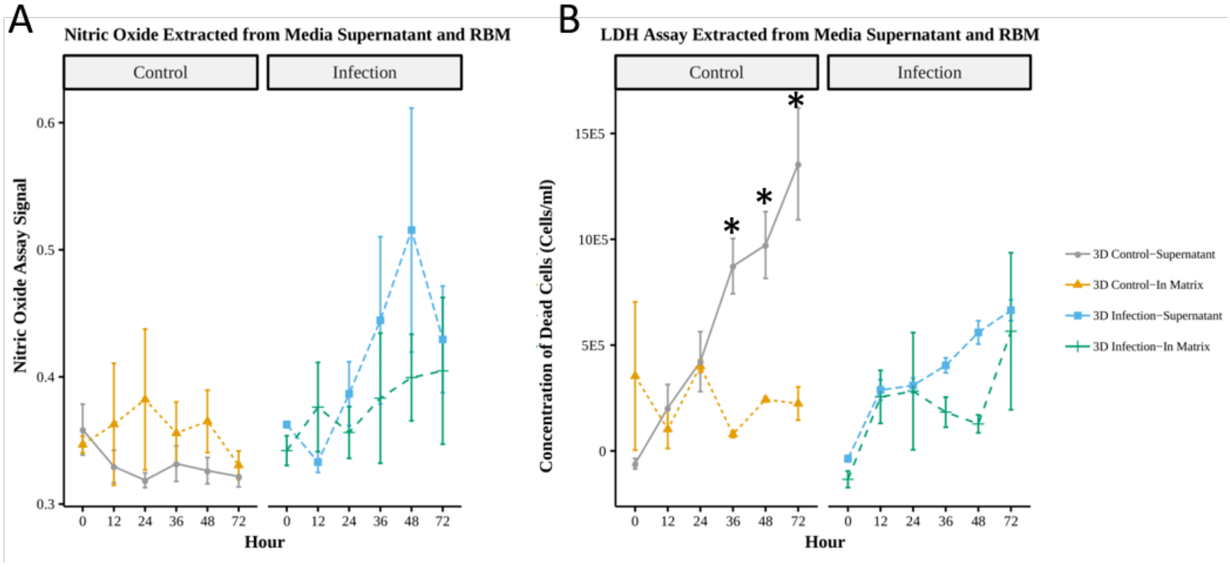

Figure S 4: Comparison of Nitric Oxide and LDH From Media supernatant and RBM. A) No significant difference between NO signal from supernatant and RBM-dilution\(possibly due to high error due to low signal as a result of microplate sensitivity); B) significant difference (p<.05) between LDH detected in supernatant and RBM at later timepoints.*

Table S 8*: Griess Assay-Signal Extracted from Media Supernatant and RBM-adjusted for dilution: Wilcoxon (n=4)*

Hour	*Control: 3D-Supernatant*	*Control: 3D-In matrix*	*p value*
**0**	0.3585	0.34675	1
**12**	0.3295	0.36275	0.885234
**24**	0.31875	0.38225	1
**36**	0.33175	0.35575	0.665006
**48**	0.32625	0.365	0.191267
**72**	0.32175	0.3305	0.885234
Hour	*Infected: 3D-Supernatant*	*Infected: 3D-In matrix*	*p value*
**0**	0.3625	0.342	0.312321
**12**	0.333	0.37625	0.312321
**24**	0.38675	0.35625	0.470486
**36**	0.4445	0.38325	0.470486
**48**	0.5155	0.3995	0.665006
**72**	0.4295	0.40475	0.665006

Table S 9*: LDH Assay Cell Death per mL Extracted from Media Supernatant and RBM: Wilcoxon (n=4)*

Hour	*Control: 3D-Supernatant*	*Control: 3D-In matrix*	*p value*
**0**	-60617.6	354040.9	1
**12**	203256	102841	0.312321
**24**	421959.1	400836.2	1
**36**	873339	78793.42	0.030383
**48**	972779.1	242902	0.030383
**72**	1356241	224053.9	0.030383
Hour	***Infected: 3D-Supernatant***	***Infected: 3D-In matrix***	***p value***
**0**	-36570	-134060	0.030383
**12**	288072.5	255575.7	1
**24**	309195.4	282223.1	0.312321
**36**	404410.9	183108	0.112351
**48**	559745.3	127538.5	0.030383
**72**	664709.8	565269.8	0.312321

3. Supplemental Results – Biochemical Response

2D and 3D infection assay Bacterial load and Effector Molecule Expression

Table S 10: 2D 3D MOI 50 CFU/mL: Log2FC Wilcoxon (n=4)

Hour	*2D*	*3D*	*p value*
**0**	0	0	-
**12**	-5.4057	-6.5588	0.47049
**24**	-7.5916	-7.3823	1
**36**	-6.5644	-8.4127	0.37676
**48**	-7.2291	-9.0334	0.595883
**72**	-8.5010	-9.3100	0.487453

Table S 11: 2D 3D MOI 50 differentials (tp1-tp0): Log2FC Wilcoxon (n=4)

Hour	*2D*	*3D*	*p value*
**12**	-5.4057	-6.5588	0.47049
**24**	-2.1858	-0.8235	0.03038

**36**	0.1209	-1.0304	0.11161
**48**	0.3625	-0.8060	0.19043
**72**	-3.1287	-1.0570	0.14891

Table S 12: Nitric Oxide Extracted from Media Supernatant 2D vs. 3D: Log2FC Signal Wilcoxon (n=4)

Hour	*2D: Control*	*3D: Control*	*p value*
**0**	0	0	-
**12**	-0.002	-0.102	0.112351
**24**	-0.083	-0.121	0.470486
**36**	-0.08	-0.109	1
**48**	0.0516	-0.115	0.470486
**72**	-0.03	-0.109	0.470486
Hour	*2D: Infection*	*3D: Infection*	*p value*
**0**	0	0	-
**12**	-0.147	-0.018	0.112351
**24**	-0.109	0.1707	0.112351
**36**	0.0134	0.2455	1
**48**	0.0333	0.533	0.312321
**72**	-0.141	0.3104	0.060602

Table S 13: Nitric Oxide Extracted from Media Supernatant Control vs Infection: Log2FC Signal Wilcoxon (n=4)

Hour	*2D: Control*	*2D: infection*	*p value*
**0**	0	0	0.112351
**12**	-0.002	-0.147	0.665006
**24**	-0.083	-0.109	0.193931
**36**	-0.08	0.0134	1
**48**	0.0516	0.0333	0.470486
**72**	-0.03	-0.141	0.112351
Hour	*3D: Control*	*3D: Infection*	*p value*
**0**	0	0	-
**12**	-0.102	-0.018	0.112351
**24**	-0.121	0.1707	0.060602
**36**	-0.109	0.2455	0.470486
**48**	-0.115	0.533	0.112351
**72**	-0.109	0.3104	0.060602

Table S 14: Differentials-Nitric Oxide Extracted from Media Supernatant 2D vs. 3D: Log2FC Signal Wilcoxon (n=4)

Hour	*2D: Control*	*3D: Control*	*p value*
**12**	-0.00151	-0.10229	0.112351
**24**	-0.08135	-0.01911	0.885234
**36**	0.00274	0.01214	0.885234
**48**	0.13170	-0.00619	0.665006
**72**	-0.08174	0.00683	0.885234
Hour	***2D: Infection***	***3D: Infection***	***p value***
**12**	-0.14739	-0.01850	0.112351
**24**	0.03819	0.18922	0.312321
**36**	0.12263	0.07477	0.665006
**48**	0.01990	0.28755	0.112351
**72**	-0.17440	-0.22261	0.885234

Table S 15: Differentials - Nitric Oxide Extracted from Media Supernatant Control vs. Infected: Log2FC Signal Wilcoxon (n=4)

Hour	*2D: Control*	*2D: Infection*	*p value*
**12**	-0.00151	-0.14739	0.112351
**24**	-0.08135	0.03819	0.885234
**36**	0.00274	0.12263	0.312321
**48**	0.13170	0.01990	0.665006
**72**	-0.08174	-0.17440	0.665006
Hour	***3D: Control***	***3D: Infection***	***p value***
**12**	-0.10229	-0.01850	0.112351
**24**	-0.01911	0.18922	0.112351
**36**	0.01214	0.07477	1
**48**	-0.00619	0.28755	0.193931
**72**	0.00683	-0.22261	0.470486

Table S 16: LDH Extracted from Media Supernatant 2D vs. 3D: Log2FC Signal Wilcoxon (n=4)

Hour	*2D: Control*	*3D: Control*	*p value*
0	0	0	-
12	0.852612	1.482648	0.193931
24	1.392919	2.184865	0.060602
36	2.14604	3.072038	0.030383
48	2.353846	3.193532	0.030383
72	2.770592	3.579989	0.030383

Hour	*2D: Infection*	*3D: Infection*	*p value*
**0**	0	0	-
**12**	1.477391	1.628312	1
**24**	1.530684	1.702693	1
**36**	1.846927	1.956242	1
**48**	2.143491	2.286088	1
**72**	2.790721	2.479411	0.193931

Table S 17: LDH Extracted from Media Supernatant Control vs. Infected: Log2FC Signal Wilcoxon (n=4)

Hour	*2D: Control*	*2D: Infection*	*p value*
**0**	0	0	-
**12**	0.852612	1.477391	0.312321
**24**	1.392919	1.530684	1
**36**	2.14604	1.846927	1
**48**	2.353846	2.143491	1
**72**	2.770592	2.790721	0.885234

Hour	*3D: Control*	*3D: Infection*	*p value*
**0**	0	0	-
**12**	1.482648	1.628312	0.885234
**24**	2.184865	1.702693	0.030383
**36**	3.072038	1.956242	0.030383
**48**	3.193532	2.286088	0.030383
**72**	3.579989	2.479411	0.030383

Table S 18: Differentials-LDH Extracted from Media Supernatant 2D vs. 3D: Log2FC Signal Wilcoxon (n=4)

Hour	*2D: Control*	*3D: Control*	*p value*
**12**	0.852612	1.482648	0.193931
**24**	0.540307	0.702217	0.665006
**36**	0.753121	0.887173	0.665006
**48**	0.207806	0.121493	0.193931
**72**	0.416747	0.386457	0.885234

Hour	*2D: Infection*	*3D: Infection*	*p value*
**12**	1.477391	1.628312	1
**24**	0.053293	0.074381	1
**36**	0.316243	0.25355	1
**48**	0.296563	0.329846	0.885234
**72**	0.647231	0.193323	0.193931

Table S 19: Differentials-LDH Extracted from Media Supernatant Control vs. Infected: Log2FC Signal Wilcoxon (n=4)

Hour	*2D: Control*	*2D: Infection*	*p value*
**12**	0.852612	1.477391	0.312321
**24**	0.540307	0.053293	0.112351
**36**	0.753121	0.316243	0.060602
**48**	0.207806	0.296563	0.665006
**72**	0.416747	0.647231	0.885234
Hour	*3D: Control*	*3D: Infection*	*p value*
**12**	1.482648	1.628312	0.885234
**24**	0.702217	0.074381	0.030383
**36**	0.887173	0.25355	0.030383
**48**	0.121493	0.329846	0.030383
**72**	0.386457	0.193323	0.112351

4. Supplemental Results – Spatiotemporal Response

Normalization of RFP intensity means

Table S 20: RFP intensity mean Trial 1 -not adjusted: Wilcoxon

Condition 1	*RFP Mean*	*Condition 2*	*RFP Mean*	*p value*
2D Control	52.78434	3D Control	55.65792	5.47E-157
2D Infection	72.69641	3D Infection	73.18344	1.16E-15
2D Control	52.78434	2D Infection	72.69641	0
3D Control	55.65792	3D Infection	73.18344	1.54E-128

Table S 21: RFP intensity mean Trial 1 adjusted: Wilcoxon

Condition 1	*RFP Mean*	*Condition 2*	*RFP Mean*	*p value*
2D Control	51.1845	3D Control	51.41812	0.573423
2D Infection	71.67664	3D Infection	70.4806	6.11E-39
2D Control	51.1845	2D Infection	71.67664	0
3D Control	51.41812	3D Infection	70.4806	1.76E-250

2D and 3D cell dynamics

Table S 22: Average Individual Cell Speed Over All Time: Wilcoxon

Condition 1	*Speed µm/s*	*Condition 2*	*Speed µm/s*	*p value*
2D Control	1.29E-03	3D Control	2.75E-04	0
2D Infection	7.61E-04	3D Infection	3.80E-04	0
2D Control	1.29E-03	2D Infection	7.61E-04	0
3D Control	2.75E-04	3D Infection	3.80E-04	4.41E-41

Table S 23: Average Individual Cell Speed 2D vs. 3D Over Time (timeframes): Wilcoxon

Hour Range	*Condition 1*	*Speed µm/s*	*Condition 2*	*Speed µm/s*	*p value*
**0-12**	2D Control	1.37E-03	3D Control	2.40E-04	2.99E-213
**12-24**	2D Control	1.28E-03	3D Control	2.25E-04	1.22E-208
**24-36**	2D Control	1.32E-03	3D Control	2.98E-04	1.35E-150
**36-48**	2D Control	1.31E-03	3D Control	3.66E-04	5.31E-82
**48-72**	2D Control	1.24E-03	3D Control	3.05E-04	1.88E-76
**0-12**	2D Infection	1.05E-03	3D Infection	4.86E-04	2.27E-47
**12-24**	2D Infection	1.04E-03	3D Infection	4.70E-04	7.94E-73
**24-36**	2D Infection	7.60E-04	3D Infection	3.69E-04	3.87E-86
**36-48**	2D Infection	6.60E-04	3D Infection	3.54E-04	9.12E-76
**48-72**	2D Infection	5.84E-04	3D Infection	3.08E-04	6.10E-127

Table S 24: Average Individual Cell Speed Control vs. Infected Over Time (timeframes): Wilcoxon

Hour Range	*Condition 1*	*Speed µm/s*	*Condition 2*	*Speed µm/s*	*p value*
**0-12**	2D Control	1.37E-03	2D Infection	1.05E-03	2.93E-21
**12-24**	2D Control	1.28E-03	2D Infection	1.04E-03	2.48E-12
**24-36**	2D Control	1.32E-03	2D Infection	7.60E-04	5.56E-57
**36-48**	2D Control	1.31E-03	2D Infection	6.60E-04	1.10E-78
**48-72**	2D Control	1.24E-03	2D Infection	5.84E-04	3.07E-156
**0-12**	3D Control	2.40E-04	3D Infection	4.86E-04	1.51E-34
**12-24**	3D Control	2.25E-04	3D Infection	4.70E-04	1.87E-41
**24-36**	3D Control	2.98E-04	3D Infection	3.69E-04	0.000244
**36-48**	3D Control	3.66E-04	3D Infection	3.54E-04	0.403125
**48-72**	3D Control	3.05E-04	3D Infection	3.08E-04	0.829769

Table S 25: Average Individual Cell Acceleration Over All Time: Wilcoxon

Condition 1	*Acceleration µm/s²*	*Condition 2*	*Acceleration µm/s²*	*p value*
2D Control	5.81E-08	3D Control	2.49E-08	1.29E-225
2D Infection	4.98E-08	3D Infection	3.28E-08	1.72E-167
2D Control	5.81E-08	2D Infection	4.98E-08	4.94E-21
3D Control	2.49E-08	3D Infection	3.28E-08	1.67E-60

Table S 26: Average Individual Cell Acceleration 2D vs. 3D Over Time (timeframes): Wilcoxon

Hour Range	*Condition 1*	*Acceleration µm/s²*	*Condition 2*	*Acceleration µm/s²*	*p value*
**0-12**	2D Control	6.00E-08	3D Control	1.95E-08	1.14E-65
**12-24**	2D Control	5.80E-08	3D Control	2.38E-08	1.97E-62
**24-36**	2D Control	5.70E-08	3D Control	2.94E-08	2.85E-36
**36-48**	2D Control	5.69E-08	3D Control	2.71E-08	2.20E-29
**48-72**	2D Control	5.89E-08	3D Control	2.71E-08	3.31E-26
**0-12**	2D Infection	4.74E-08	3D Infection	2.91E-08	2.74E-12
**12-24**	2D Infection	5.05E-08	3D Infection	3.41E-08	1.24E-15
**24-36**	2D Infection	5.21E-08	3D Infection	3.23E-08	5.77E-46
**36-48**	2D Infection	4.96E-08	3D Infection	3.39E-08	3.36E-37
**48-72**	2D Infection	4.90E-08	3D Infection	3.32E-08	4.67E-66

Table S 27: Average Cell Acceleration Control vs. Infected Over Time (timeframes): Wilcoxon

Hour Range	*Condition 1*	*Acceleration µm/s²*	*Condition 2*	*Acceleration µm/s²*	*p value*
**0-12**	2D Control	6.00E-08	2D Infection	4.74E-08	2.46E-11
**12-24**	2D Control	5.80E-08	2D Infection	5.05E-08	4.86E-06
**24-36**	2D Control	5.70E-08	2D Infection	5.21E-08	0.095358
**36-48**	2D Control	5.69E-08	2D Infection	4.96E-08	0.002894
**48-72**	2D Control	5.89E-08	2D Infection	4.90E-08	6.93E-08
**0-12**	3D Control	2.38E-08	3D Infection	2.91E-08	1.75E-15
**12-24**	3D Control	2.94E-08	3D Infection	3.41E-08	2.45E-13
**24-36**	3D Control	2.71E-08	3D Infection	3.23E-08	0.000152
**36-48**	3D Control	2.71E-08	3D Infection	3.39E-08	4.29E-06
**48-72**	3D Control	2.38E-08	3D Infection	3.32E-08	3.24E-07

Table S 28: Average Individual Cell Directedness Over All Time: Wilcoxon

Condition 1	*Directedness*	*Condition 2*	*Directedness*	*p value*
2D Control	0.312	3D Control	0.189	9.36E-200
2D Infection	0.242	3D Infection	0.164	6.59E-207
2D Control	0.312	2D Infection	0.242	1.85E-152
3D Control	0.189	3D Infection	0.164	5.69E-35

Table S 29: Average Individual Cell Directedness 2D vs. 3D Over Time (timeframes): Wilcoxon

**Hour Range**	Condition 1	Directedness	Condition 2	Directedness	p value
**0-12**	2D Control	0.428	3D Control	0.364	6.32E-18
**12-24**	2D Control	0.317	3D Control	0.153	6.06E-108
**24-36**	2D Control	0.297	3D Control	0.122	5.53E-112
**36-48**	2D Control	0.298	3D Control	0.121	1.44E-67
**48-72**	2D Control	0.278	3D Control	0.128	2.03E-44
**0-12**	2D Infection	0.417	3D Infection	0.386	7.14E-05
**12-24**	2D Infection	0.303	3D Infection	0.202	5.23E-44
**24-36**	2D Infection	0.242	3D Infection	0.148	9.45E-16
**36-48**	2D Infection	0.205	3D Infection	0.123	1.91E-108
**48-72**	2D Infection	0.174	3D Infection	0.084	2.54E-167

Table S 30: Average Cell Directedness Control vs. Infected Over Time (timeframes): Wilcoxon

**Hour Range**	Condition 1	Directedness	Condition 2	Directedness	p value
**0-12**	2D Control	0.428	2D Infection	0.417	0.088243
**12-24**	2D Control	0.317	2D Infection	0.303	0.04272
**24-36**	2D Control	0.297	2D Infection	0.242	3.39E-18
**36-48**	2D Control	0.298	2D Infection	0.205	8.08E-45
**48-72**	2D Control	0.278	2D Infection	0.174	2.30E-104
**0-12**	3D Control	0.364	3D Infection	0.386	0.000579
**12-24**	3D Control	0.153	3D Infection	0.202	1.30E-32
**24-36**	3D Control	0.122	3D Infection	0.148	4.41E-13
**36-48**	3D Control	0.121	3D Infection	0.123	0.308664
**48-72**	3D Control	0.128	3D Infection	0.084	2.64E-07

Table S 31: Average Individual Cell Volume Over All Time: Wilcoxon

Condition 1	*Volume μm^3*	*Condition 2*	*Volume μm^3*	*p value*
2D Control	3.73E+03	3D Control	2.36E+03	0
2D Infection	3.80E+03	3D Infection	3.03E+03	3.99E-285
2D Control	3.73E+03	2D Infection	3.80E+03	4.04E-08
3D Control	2.36E+03	3D Infection	3.03E+03	6.11E-132

Table S 32: Average Individual Cell Volume 2D vs. 3D Over Time (timeframes): Wilcoxon

**Hour Range**	Condition 1	Volume μm^3	Condition 2	Volume μm^3	p value
**0-12**	2D Control	3.52E+03	3D Control	2.35E+03	2.61E-74
**12-24**	2D Control	3.63E+03	3D Control	2.41E+03	1.39E-87
**24-36**	2D Control	3.71E+03	3D Control	2.40E+03	1.04E-81
**36-48**	2D Control	3.74E+03	3D Control	2.30E+03	9.75E-79
**48-72**	2D Control	3.89E+03	3D Control	2.29E+03	1.32E-73
**0-12**	2D Infection	3.36E+03	3D Infection	2.79E+03	0.001391
**12-24**	2D Infection	3.97E+03	3D Infection	3.12E+03	3.96E-06
**24-36**	2D Infection	4.02E+03	3D Infection	3.10E+03	5.10E-15
**36-48**	2D Infection	3.89E+03	3D Infection	3.01E+03	8.16E-06
**48-72**	2D Infection	3.67E+03	3D Infection	3.05E+03	0.018879

Table S 33: Average Individual Cell Volume Control vs. Infected Over Time (timeframes): Wilcoxon

**Hour Range**	Condition 1	Volume μm^3	Condition 2	Volume μm^3	p value
**0-12**	2D Control	3.52E+03	2D Infection	3.36E+03	8.36E-25
**12-24**	2D Control	3.63E+03	2D Infection	3.97E+03	1.09E-44
**24-36**	2D Control	3.71E+03	2D Infection	4.02E+03	2.58E-76
**36-48**	2D Control	3.74E+03	2D Infection	3.89E+03	1.38E-70
**48-72**	2D Control	3.89E+03	2D Infection	3.67E+03	1.95E-81
**0-12**	3D Control	2.35E+03	3D Infection	2.79E+03	7.47E-07
**12-24**	3D Control	2.41E+03	3D Infection	3.12E+03	1.89E-22
**24-36**	3D Control	2.40E+03	3D Infection	3.10E+03	3.40E-25
**36-48**	3D Control	2.30E+03	3D Infection	3.01E+03	2.70E-36
**48-72**	3D Control	2.29E+03	3D Infection	3.05E+03	2.99E-39

Temporally correlated characteristics vary with infection and environment

3D Infection has a high positive correlation between nitric oxide and hours in culture (0.7); all other conditions have a low correlation with time (< +/-0.2). LDH and hours in culture maintains a high positive correlation regardless of condition (>0.9, p <.05). Cell speed and hours in culture presents a high positive correlation in 3D control condition (0.77) all other conditions have a high negative (<-0.7) correlation but only 3D and 2D infection are significant (p<.05). Acceleration and hours in culture presents a minimal correlation in 2D (<+/-0.3), but both 3D control and infection present a high positive correlation (>0.5). Directedness and hours in culture maintains a high negative correlation regardless of condition (<-0.6), but only shows significance in 2D infection (p<.05). Cell volume and hours in culture shows a high positive correlation in 2D control (0.95, p <.05) and 3D control presents with a high negative correlation (-0.53). 2D and 3D Infected conditions both have low positive correlations (<0.5). CFU and hours in culture have a high negative correlation in both 2D and 3D infection (<-0.8), but only shows significance in 3D infection (p<.05). The adjusted RFP mean and hours in culture presents with a high negative correlation in all conditions even after the field-based normalization (<-0.7) but significance is only shown in 2D controls (p<.05).

Non-Windowed Cell Dynamic Data

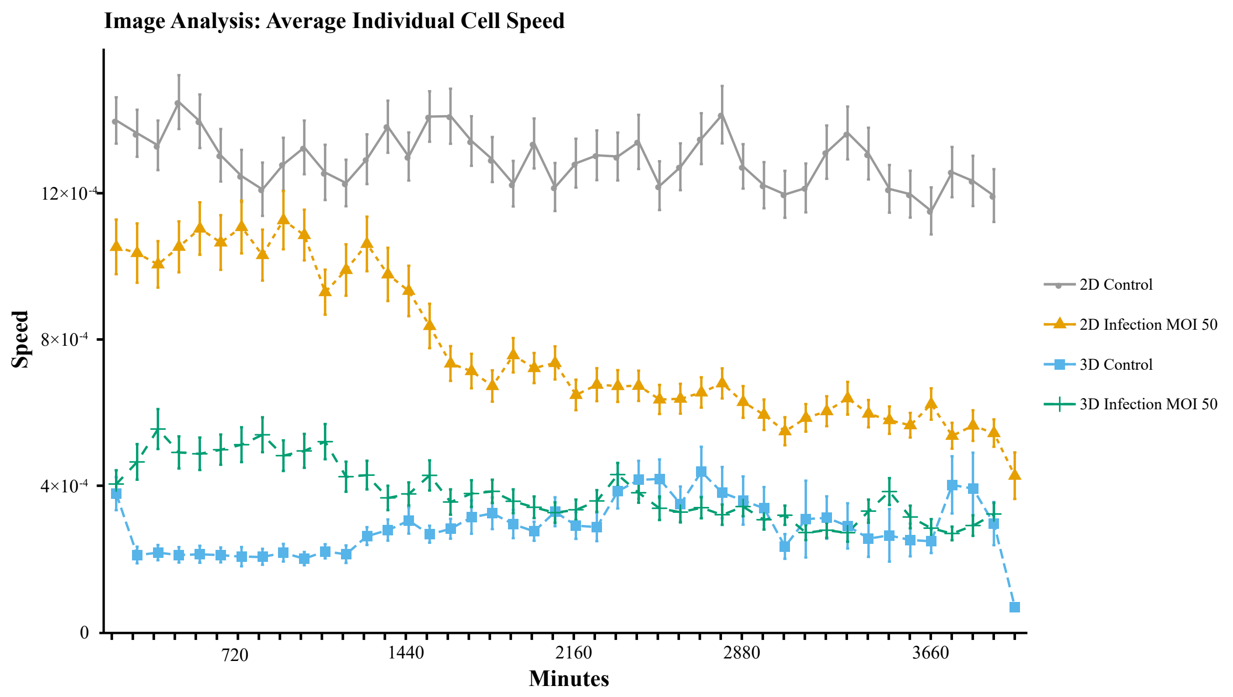

Figure S 5: Image analysis of individual cell speed of gfpBMDM infected with mCherry M.smegmatis in 2D and 3D culture conditions over 72 hours. Minutes rounded to nearest whole 60 minutes to account for time delays between imaging points.

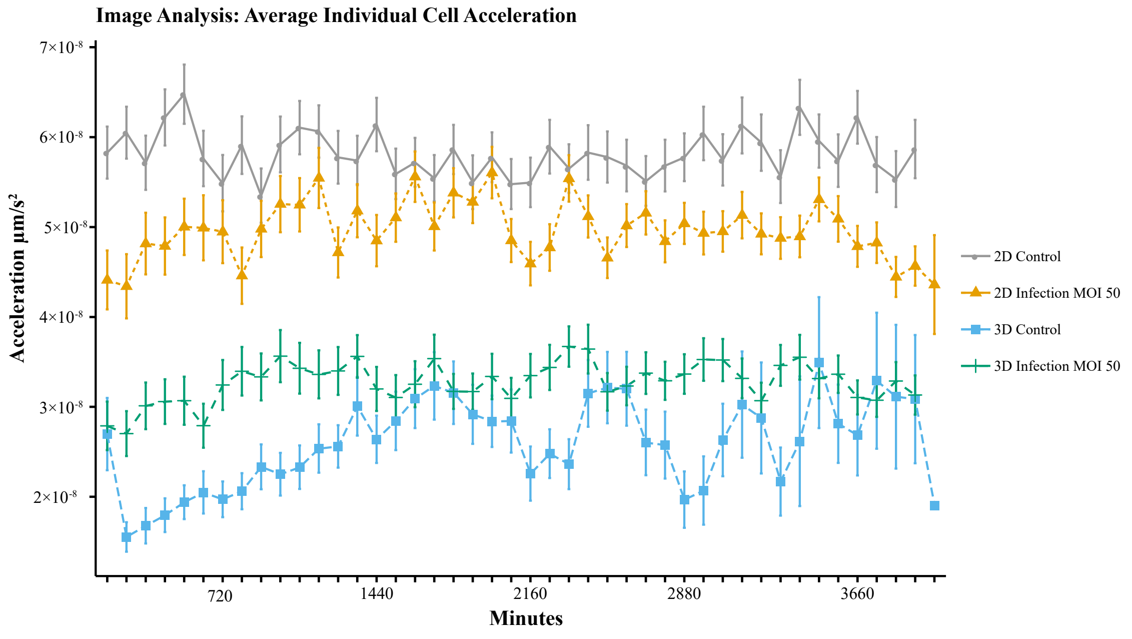

Figure S 6: Image analysis of individual cell acceleration of gfpBMDM infected with mCherry M.smegmatis in 2D and 3D culture conditions over 72 hours. Minutes rounded to nearest whole 60 minutes to account for time delays between imaging points.

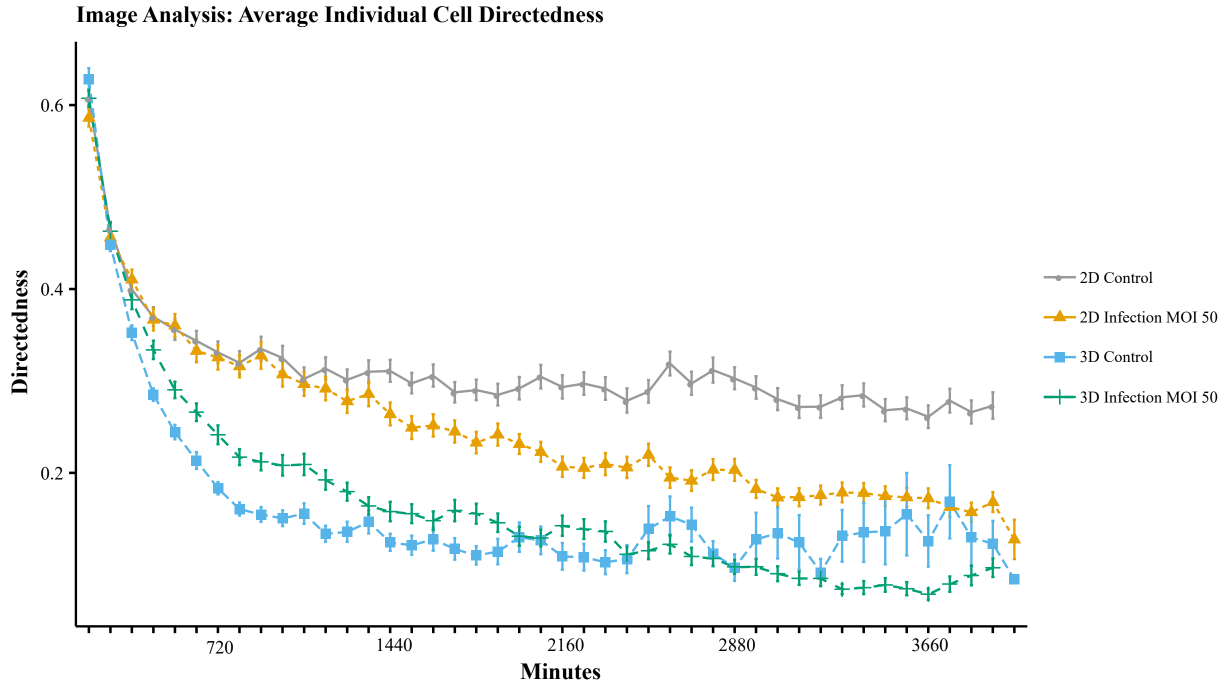

Figure S 7: Image analysis of individual cell directedness of gfpBMDM infected with mCherry M.smegmatis in 2D and 3D culture conditions over 72 hours. Minutes rounded to nearest whole 60 minutes to account for time delays between imaging points.

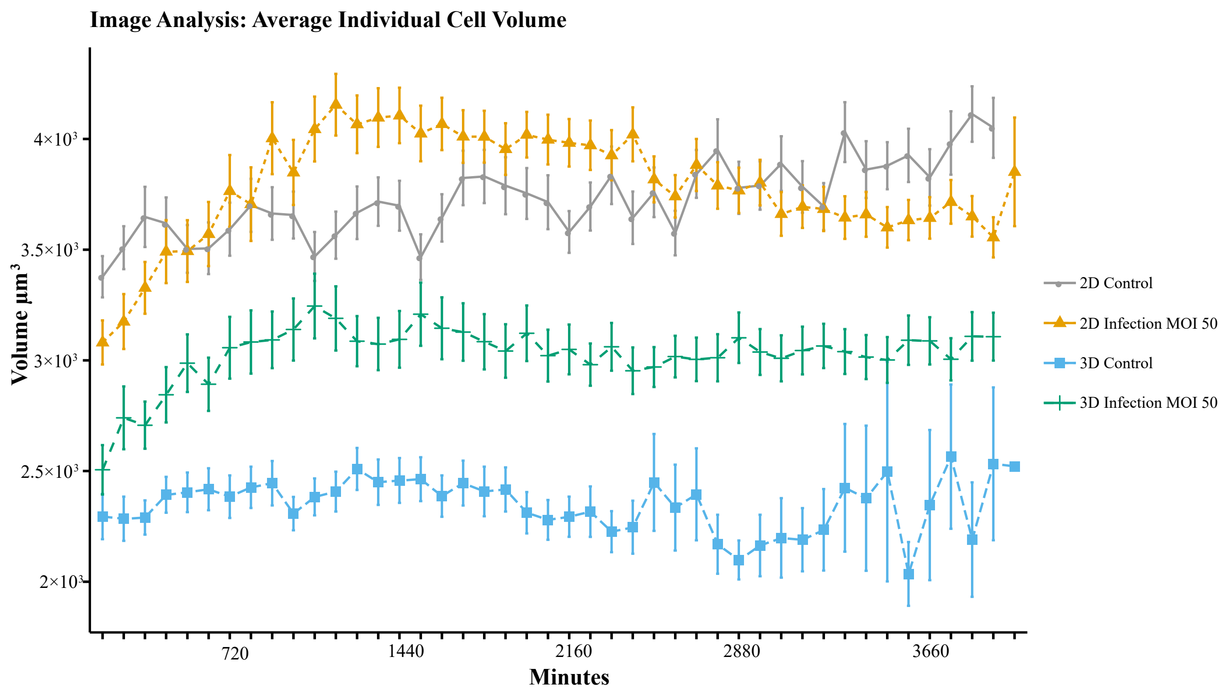

Figure S 8: Image analysis of individual cell volume of gfpBMDM infected with mCherry M.smegmatis in 2D and 3D culture conditions over 72 hours. Minutes rounded to nearest whole 60 minutes to account for time delays between imaging points.

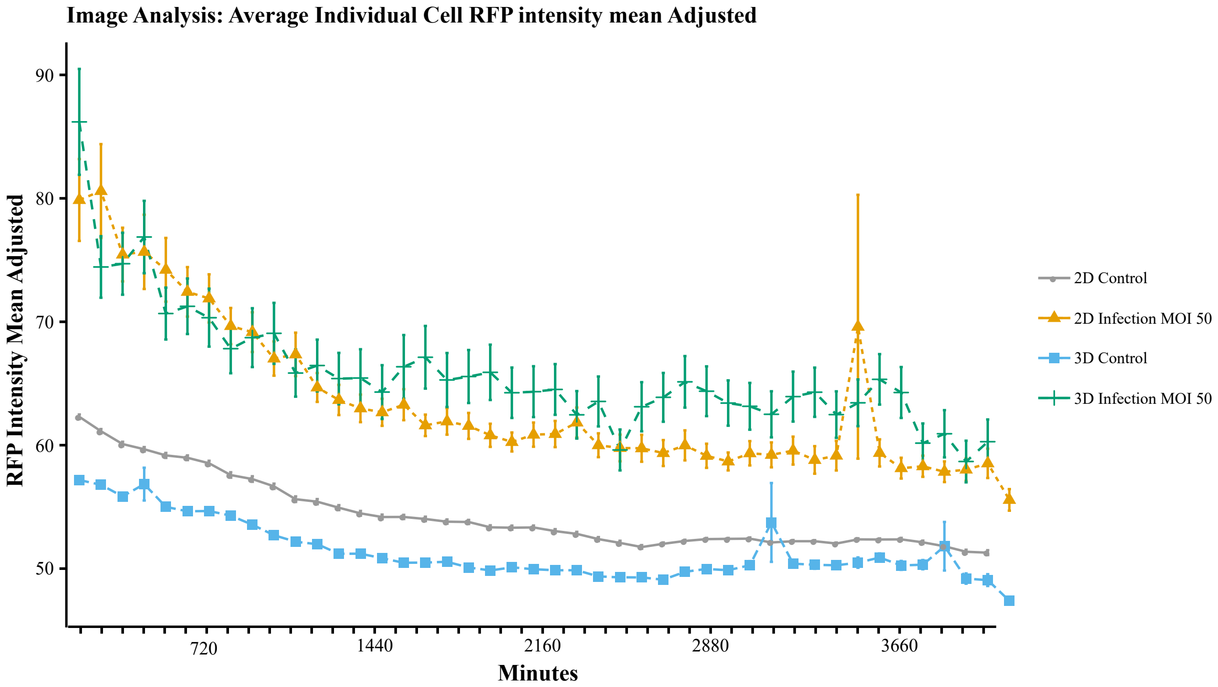

Figure S 9: Image analysis of individual cell RFP Intensity mean (adjusted) of gfpBMDM infected with mCherry M.smegmatis (trial 1 and 2 combined) in 2D and 3D culture conditions over 72 hours. Minutes rounded to nearest whole 60 minutes to account for time delays between imaging points.

5. References

[1] M. E. Gough, E. A. Graviss, T.-A. Chen, E. M. Obasi, and E. E. May, "Compounding effect of vitamin D3 diet, supplementation, and alcohol exposure on macrophage response to mycobacterium infection," *Tuberculosis*, p. S1472979219301490, Apr. 2019.

[2] F. M. Marim, T. N. Silveira, D. S. Lima, and D. S. Zamboni, "A Method for Generation of Bone Marrow-Derived Macrophages from Cryopreserved Mouse Bone Marrow Cells," *PLoS ONE*, vol. 5, no. 12, Dec. 2010.

[3] M. E. Gough, E. A. Graviss, and E. E. May, "The dynamic immunomodulatory effects of vitamin D3 during Mycobacterium infection," *Innate Immun.*, vol. 23, no. 6, pp. 506–523, Aug. 2017.

[4] L. D. Loose, "Characterization of Macrophage Dysfunction in Rodent Malaria," J. Leukoc. Biol., vol. 36, no. 6, pp. 703–718, 1984.

[5] C. L. Sershen, S. J. Plimpton, and E. E. May, "Oxygen Modulates the Effectiveness of Granuloma Mediated Host Response to Mycobacterium tuberculosis: A Multiscale Computational Biology Approach," Front. Cell. Infect. Microbiol., vol. 6, Feb. 2016.
